# The Tacrine‐Induced Endoplasmic Reticulum Stress in AChE‐Expressed Cells Leads to Improper Assembly and Transport of the Oligomeric Enzyme: Reversal by Trehalose

**DOI:** 10.1111/jnc.70178

**Published:** 2025-08-05

**Authors:** Xiaoyang Wang, Yingjie Xia, Maggie Suisui Guo, Jiahui Wu, Ajiaikebaier Dilidaer, Jin Gao, Tina Tingxia Dong, Yue Zhu, Karl Wah Keung Tsim

**Affiliations:** ^1^ Division of Life Science, Center for Chinese Medicine and State Key Laboratory of Molecular Neuroscience The Hong Kong University of Science and Technology Hong Kong China; ^2^ Shenzhen Key Laboratory of Edible and Medicinal Bioresources The Hong Kong University of Science and Technology Shenzhen China; ^3^ Department of Neurobiology and Cellular Biology Xuzhou Medical University Xuzhou China; ^4^ Jiangsu Key Laboratory for High Technology Research of TCM Formulae and Jiangsu Collaborative Innovation Center of Chinese Medicinal Resources Industrialization Nanjing University of Chinese Medicine Nanjing China

**Keywords:** acetylcholinesterase inhibitor, cholinesterase, ER stress, tacrine, trehalose

## Abstract

Alzheimer's disease (AD) is the most common dementia with progressive loss of cognitive functions. Acetylcholinesterase (AChE) inhibitors have been approved as conventional pharmacotherapies for AD. Tacrine was the first AChE inhibitor introduced into clinics for AD; however, it was withdrawn from use in 2013 because of safety concerns. In cultured neurons, as well as in mice, tacrine was found to induce endoplasmic reticulum (ER) stress and finally lead to cell apoptosis: the event was triggered by binding the inhibitor to the intracellular enzyme serving as a pharmacological chaperone. Trehalose, a known ER stress reducer, was shown here to ameliorate the ER stress induced by tacrine in AChE‐overexpressed NG108‐15 cells, with the increased level of C/EBP homologous protein (CHOP) and phosphorylated eukaryotic initiation factor 2 alpha (p‐eIF2α). In tetrameric G4 AChE overexpressed cells, the tacrine‐exposed cultures revealed considerable G1/G2 forms of AChE accumulated in the ER fraction, whereas the treatment of trehalose decreased the accumulation of G1/G2 AChE. Meanwhile, trehalose reduced the ER stress induced by other AChE inhibitors, for example, lycobetaine, bis(3)‐cognitin, daurisoline, and dauricine, in the cultured neuronal cells. Besides, this tacrine‐induced ER stress was identified in all AChE isoforms, as well as in butyrylcholinesterase (BChE) expressing cells. Thus, we proposed that the AChE inhibitors, particularly tacrine, could act as ‘chemical/pharmacological chaperones’ during AChE biosynthesis in the ER, disrupting the proper folding of AChE in neurons as a result of ER stress. Trehalose possesses the ability to relieve ER stress by promoting the proper assembly of AChE. The results provide guidance for the drug design and discovery of AChE inhibitors for AD treatment.

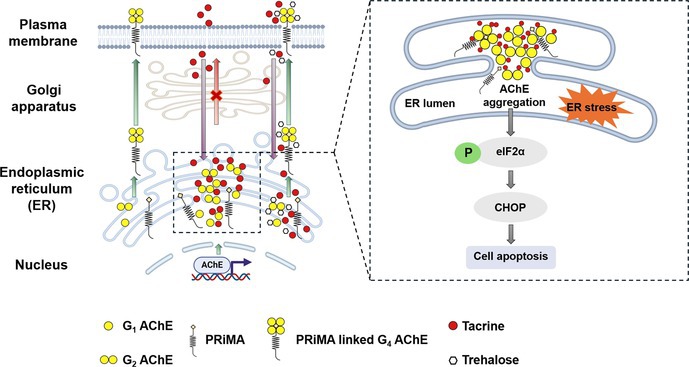

## Graphical abstract

Acetylcholinesterase (AChE) inhibitors are a class of drugs to treat Alzheimer's disease (AD). Our study reveals that some AChE inhibitors, for example, tacrine, disrupt the proper assembly and transport of oligomeric forms of AChE, resulting in endoplasmic reticulum (ER) stress. The ER stress ultimately leads to cell apoptosis. This tacrine‐induced ER stress could happen in all AChE isoforms, as well as in butyrylcholinesterase (BChE) expressing cells. Notably, trehalose, a natural non‐reducing disaccharide, possesses the ability to relieve the ER stress by promoting the proper assembly of AChE. The results provide guidance for safer design of AChE inhibitors for AD treatment.

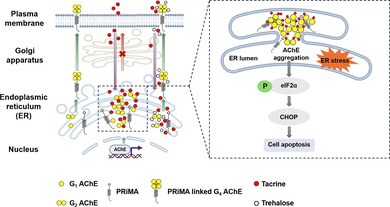

**Abbreviations**
ACh: acetylcholine
AChE: acetylcholinesterase
AD: Alzheimer's disease
BChE: butyrylcholinesterase
CHOP: C/EBP homologous protein
DAPI: 4′,6‐diamidino‐2‐phenylindole
eIF2α: Eukaryotic initiation factor 2 alpha
ER: endoplasmic reticulum
GPI: glycosylphosphatidylinositol
P‐eIF2α: phosphorylated eukaryotic initiation factor 2 alpha
PRiMA: proline‐rich membrane anchor
RRID: Research Resource Identifier (see scicrunch.org)
UPR: Unfolded protein response

## 1 Introduction

Cholinesterase widely exists in all vertebrates and has two major classes: acetylcholinesterase (AChE) and butyrylcholinesterase (BChE) (Massoulié 2002). AChE hydrolyzes the neurotransmitter acetylcholine (ACh) rapidly at cholinergic synapses, which is essential in controlling neurotransmission. The *AChE* gene encodes three isoform types: AChE_R_, AChE_H_, and AChE_T_ (Legay et al. 1995; Massoulié et al. 2005). AChE_T_ is the major isoform being expressed in the brain and muscle. In the brain, AChE_T_ is linked with a proline‐rich membrane anchor (PRiMA) to anchor the enzyme to the cell membrane, specifically at cholinergic synapses (Xie et al. 2010). In muscles, the AChE_T_ tetramer is linked with a collagen tail, namely ColQ (Tsim et al. 1988; Krejci et al. 1991). In both neural and muscle cells, the activity of AChE is crucial in maintaining the balance of neurotransmission and muscle contraction, ensuring precise and controlled signaling within the nervous system and proper muscle functions. AChE_H_ subunits produce glycosylphosphatidylinositol (GPI)‐anchored dimers, which are usually expressed in blood cells (lymphocytes and erythrocytes) (Massoulié et al. 1998b). BChE_T_, the only variant of BChE, could be linked with PRiMA or ColQ. In vertebrate neuromuscular junctions, these cholinesterase isoforms play important roles in regulating acetylcholine levels and are essential for proper neuromuscular functions (Girard et al. 2007; Leung et al. 2009; Massoulié and Millard 2009; Tsim et al. 2010).

Alzheimer's disease (AD) is the most common form of dementia with progressive loss of cognitive functions. Loss of cholinergic tone and ACh level in the brain was hypothesized to be responsible for the cognitive decline (Davies and Maloney 1976). The enzymatic activity of AChE was found to be enhanced within and around amyloid plaques in the brain of AD patients (Ulrich et al. 1990; Morán et al. 1993), increasing the neurotoxicity of Aβ fibrils (Alvarez et al. 1998), and leading to a higher breakdown of ACh in the brain, that is, total reduction of ACh in the brain and the demise of the neuron. Hence, AChE inhibitors, that is, galantamine, donepezil, and rivastigmine, have been approved as conventional pharmacotherapies for AD; these inhibitors can slow down cognitive deterioration and improve behavioral and daily living conditions in clinical usage (Giacobini 2000). Tacrine is the first AChE inhibitor introduced into clinical use as a therapy for AD, improving mental functioning in patients with mild‐to‐moderate AD. Tacrine acts by reversibly inhibiting the metabolism of ACh, prolonging the level of ACh in the cerebral cortex (Crismon 1994). However, tacrine was withdrawn from clinical use in 2013 because of safety concerns (Watkins et al. 1994; Blackard et al. 1998).

In eukaryotic cells, the endoplasmic reticulum (ER) is crowded with molecular chaperones and protein‐modification enzymes, helping the protein to fold properly. When the proteins cannot be properly folded in the ER, the unfolded or misfolded proteins are accumulated in the ER, leading to ER stress (Schröder and Kaufman 2005). Eukaryotic cells have developed several signaling transductions to sense and respond to ER stress, that is, the unfolded protein response (UPR) (Hetz et al. 2020). UPR has three ways to restore the normal functions of the cell: (i) decreasing the protein translation; (ii) eliminating the misfolded proteins; and (iii) activating the production of molecular chaperones. If these strategies cannot relieve the ER stress, the UPR leads to cell apoptosis. Several lines of evidence suggest that ER stress may be another essential mechanism in drug‐induced adverse effects (Foufelle and Fromenty 2016). Trehalose, a natural non‐reducing disaccharide, consists of two glucose molecules, which has been proven to suppress inflammation, promote molecular chaperone production, and to retrieve the ER stress (Jain and Roy 2009; Tang et al. 2017). Tacrine, as well as other AChE inhibitors, was found to induce ER stress and lead to apoptosis in neurons (Liu et al. 2021): these events might explain the adverse effects, as induced by these inhibitors. Here, the restoration of tacrine‐induced ER stress by application of trehalose was determined.

## 2 Materials and Methods

### 2.1 Materials

Dulbecco's modified Eagle medium (DMEM), fetal bovine serum (FBS), and other reagents for cell culture were purchased from Thermo Fisher Scientific (Waltham, MA, USA). Antibodies were purchased from Abcam (Cambridge, UK), Cell Signaling Technology (Danvers, MA, USA), and Proteintech (Wuhan, China). Tacrine was purchased from MCE (Cat# HY‐B2244, MCE, NJ, USA); trehalose was purchased from Shanghai Yuanye Bio‐Technology (Cat# S11051, Shanghai Yuanye Bio‐Technology Co. Ltd., Shanghai, China); other AChE inhibitors were purchased from Sigma‐Aldrich (St. Louis, MO, USA); and synthetic AChE inhibitors, bis(3)‐cognitin were from Prof. Yifan Han (Hong Kong PolyU) (Han et al. 2012). Dimethyl sulfoxide (DMSO; Cat# 472301), thapsigargin (Cat# T9033), and other chemicals were purchased from Sigma‐Aldrich (St. Louis, MO, USA). Thapsigargin, tacrine, and other AChE inhibitors were dissolved in DMSO.

### 2.2 Cell Culture

Mouse neuroblastoma X rat glioma hybrid cells (NG108‐15, RRID:CVCL_0464) and human embryonic kidney fibroblast cells (HEK293T, RRID:CVCL_0063) were purchased from American Type Culture Collection (ATCC; Manassas, VA, USA). The cells were maintained in high glucose Dulbecco's Modified Eagle Medium (Cat# 12800017) supplemented with 1% (v/v) penicillin/streptomycin (10 000 U/mL, Cat# 15140122) and 10% fetal bovine serum (Cat# A5256701) at 37°C in an incubator with a water‐saturated atmosphere and 5% CO_2_. All culture reagents and solutions were purchased from Thermo Fisher Scientific. The maximum number of passages of NG108‐15 and HEK293T cell lines was 30. The cell lines were last authenticated through STR authentication on 2025/06/25. These two cell lines are not listed as commonly misidentified cell lines by the International Cell Line Authentication Committee (ICLAC).

### 2.3 DNA Construct and Transfection

The cDNA encoding human AChE_T_, PRiMA, AChE_H_, and human *ACHE* promoter tagged with luciferase (pAChE‐Luc) was described previously (Chen et al. 2011; Luk et al. 2012; Liu et al. 2021). The cDNA encoding BChE_T_ tagged with a FLAG epitope at the N terminus was subcloned into a pcDNA4 vector under a CMV promoter. The cDNA encoding full‐length human ColQ tagged with FLAG was ordered from Sino Biological (Cat# HG19876‐CF, Sino Biological, Beijing, China). Transient transfection of NG108‐15 and HEK293T cells was performed using a jetPRIME Transfection kit (Cat# 101000046, Polyplus, France) according to the manufacturer's instructions. In brief, the mixture of DNA constructs with reagents was added to the cells and incubated for 4 h in an incubator, followed by a fresh culture medium replacement.

### 2.4 Luciferase Assay

Luciferase assay was performed using Luciferase Assay System (Cat# E4550, Promega, Madison, WI, USA). In brief, cells were lysed with 1X passive lysis buffer diluted from 5X stock (Cat# E1941, Promega). Afterward, cells were centrifuged at 12 000 × g for 2 min at 4°C. The supernatant was then normalized using a protein assay and utilized for the luciferase assay. The luminescent reaction was quantified in a GloMax 96 Microplate Luminometer, and the activity was expressed as a fold of untreated controls.

### 2.5 AChE/BChE Enzymatic Assay

AChE/BChE enzymatic activity was determined based on the Ellman assay with minor modifications. The cell lysates were extracted by low salt lysis buffer containing 0.25 M NaCl, 10 mM HEPES pH 7.5, 1 mM EDTA, 1 mM EGTA, supplemented with 0.5% Triton X‐100, 2.5 mM benzamidine, 10 μg/mL aprotinin, and 10 μg/mL leupeptin. Then the lysate was added to a reaction mixture containing tetraisopropyl pyrophosphoramide (0.1 mM, iso‐OMPA, Cat# 434760, Sigma‐Aldrich) or 1,5‐Bis(4‐allyldimethylammoniumphenyl)pentan‐3‐one dibromide (0.05 mM, BW 284c51, Cat# A9013, Sigma‐Aldrich), 0.5 mM 5,5‐dithiobis‐2‐nitrobenzoic acid (DTNB), 0.625 mM acetylthiocholine/butyrylthiocholine, 80 mM disodium hydrogen phosphate, and 80 mM sodium phosphate at pH 7.4. Absorbance unit per minute per gram of protein was measured to indicate enzyme activity. The absorbance was measured at 410 nm.

### 2.6 Sucrose Density Gradient Assay

As previously described, various molecular forms of AChE were separated by sucrose density gradient assay (Xie et al. 2010). Total proteins were extracted by low‐salt lysis buffer supplemented with 0.5% Triton X‐100, 2.5 mM benzamidine, 10 μg/mL aprotinin, and 10 μg/mL leupeptin. Alkaline phosphatase (ALP, 6.1 S; Cat# 04571363103, Roche, Basel, Switzerland) and β‐galactosidase (16 S; Cat# 11184024103, Roche), employed as internal sedimentation markers, were mixed with 200 μL protein lysates (1 μg/μL). The mixture was then loaded onto continuous sucrose gradients (5%–20%) in 13.2 mL polyallomer tubes (Cat# C14293, Beckman Coulter, Brea, CA, USA). The tubes with gradients were centrifuged at 38000 rpm in a SW41 Ti rotor (Cat# 331362, Beckman Coulter) for 16 h at 4°C. About 45 fractions were then collected from the bottom to the top of the tubes. ALP, β‐galactosidase, and AChE activities were determined, reflecting the relative expression of different AChE isoforms.

### 2.7 Cell Lysates and Western Blotting Analyses

The cell lysates were extracted by low‐salt lysis buffer or high‐salt lysis buffer containing 0.25 M/0.5 M NaCl, 10 mM HEPES, pH 7.5, 1 mM EDTA, 1 mM EGTA, supplemented with 0.5% Triton X‐100, 2.5 mM benzamidine, 10 μg/mL aprotinin, and 10 μg/mL leupeptin. After adding the lysis buffer to the cells, they were subjected to agitation for 30 min at 4°C, followed by centrifugation at 13 200 rpm, 4°C for 15 min. The whole cell lysates were found in the supernatants. Bradford assay (Cat# 5000006, Bio‐Rad Laboratories, Hercules, CA) was performed to determine protein concentration in each sample. Samples containing equal amounts of total protein were treated with a direct lysis buffer, with or without 100 mM β‐mercaptoethanol, and boiled at 96°C for 15 min. The proteins (25–40 μg) were loaded onto 8%/10% running gel and 4% stacking gel of SDS‐polyacrylamide gel. The proteins were transferred to the nitrocellulose membrane (Cat# 66485, Pall Corporation, Mexico) after the electrophoresis separation in 1× SDS‐PAGE running buffer. The primary antibodies, incubated overnight at 4°C, were as follows: anti‐AChE antibody (1:1000; Cat# 183591, Abcam, Cambridge, UK, RRID:AB_2857345), anti‐CHOP antibody (1:1000; Cat# 2895, Cell Signaling Technology, Danvers, MA, USA RRID:AB_2089254), anti‐α‐tubulin antibody (1:1000; Cat# 3873, Cell Signaling Technology, RRID:AB_1904178), anti‐eIF2α antibody (1:1000; Cat# 9722, Cell Signaling Technology, AB_2230924), anti‐phospho‐eIF2alpha (Ser51) antibody (1:1000; Cat# 9721, Cell Signaling Technology, RRID:AB_330951), anti‐BChE antibody (1:1000; Cat# AF9024, R&D Systems, MN, USA, RRID:AB_3076728), anti‐sodium potassium ATPase antibody (1:1000; Cat# ab76020, Abcam, RRID:AB_1310695), anti‐calnexin antibody (1:1000; Cat# ab22595, Abcam, RRID:AB_2069006), and DYK DDD DK tag (binds to FLAG tag epitope) antibody (1:1000, Cat# 66008‐4‐Ig, Proteintech, RRID:AB_2918475). The enhanced chemiluminescence method was used to visualize the immune complexes in strictly standardized conditions. The intensities of bands were quantified by Image Laboratory 6.1 (Bio‐Rad, RRID:SCR_014210). α‐Tubulin served as an internal control to calculate the expression of target proteins.

### 2.8 Immunofluorescence Staining

NG108‐15 cells, or HEK293T cells, seeded onto poly‐L‐lysine‐coated coverslips, were washed with phosphate‐buffered saline (PBS) before being fixed with 4% paraformaldehyde for 20 min. Then, the cells were washed three times and blocked by 5% bovine serum albumin, with or without 0.1% Triton X‐100, for 1 h at room temperature. After blocking, cells were washed three times by PBS and followed by being stained with AChE antibody (1:200, Cat# ab183591, or ab2803, Abcam) or DYK DDD DK tag (binding to FLAG tag epitope) antibody (1:200, Cat# 66008‐4‐Ig, Proteintech), overnight at 4°C. After washing three times by PBS, the cells were stained with anti‐rabbit IgG (H + L), (Alexa Fluor 647 conjugate) (1:200; Cat# ab150075, Abcam, RRID:AB_2752244) or anti‐mouse IgG H&L (Alexa Fluor 647 conjugate) (1:200; Cat# ab150107, Abcam, RRID:AB_2890037) and rhodamine phalloidin (Cat# R415, Thermo Fisher Scientific) for 2 h at room temperature. After washing with PBS, the cells were mounted with ProLong Gold Antifade reagent with 4′,6‐diamidino‐2‐phenylindole (DAPI) (Cat# 9071, Cell Signaling Technology) and then subjected to visualization by SP8 confocal microscope (Leica Microsystems, Germany, RRID:SCR_018169).

### 2.9 Subcellular Fractionation

Subcellular fractionation was carried out, as previously described (Chen et al. 2011). Briefly, the cells, grown in 100‐mm dishes, were detached from confluent cultures in 1 mL homogenization buffer (10 mm HEPES, pH 7.4, 1 mM EDTA, 0.25 M sucrose, supplemented with protease inhibitors in the low‐salt lysis buffer). The cells were disrupted using 10 strokes in a Dounce homogenizer on ice. Nuclei and unbroken cells were pelleted by centrifugation at 500 × g for 10 min. The post‐nuclear supernatants were centrifuged at 80 000 × g for 1 h. The vesicle pellet was resuspended in 0.8 mL of homogenization buffer and applied on the top of linear iodixanol gradients (1%–20%; OptiPrep, Cat#07820, STEMCELL). The gradients were centrifuged in a SW41 Ti rotor at 200 000 × g for 3 h at 4°C. Sequential 1‐mL fractions were collected from the top of the gradient. The distribution of intracellular markers was examined by Western blotting with different antibodies: calnexin for the ER and Na^+^/K^+^‐ATPase for the plasma membrane (PM).

### 2.10 ER Extraction

The extraction of ER was processed as described with minimal optimization (Leiro et al. 2023). Briefly, the cells, grown in 100‐mm dishes, were detached from confluent cultures in 1 mL of homogenization buffer. The cells were disrupted using 10 strokes in a Dounce homogenizer on ice. The homogenate was centrifuged at 1000 × g for 10 min at 4°C. The supernatant was transferred to another centrifuge tube and centrifuged at 7000 × g for 15 min at 4°C. The supernatant from the previous step was transferred to the ultracentrifuge tube and centrifuged at 20 000 × g for 35 min. Then, the supernatant from the previous step was transferred to a new ultracentrifuge tube and centrifuged at 100 000 × g for 65 min. Following centrifugation, the supernatant was aspirated, and the ER pellet was resuspended in 100 μL of homogenization buffer.

### 2.11 Animal Study

Initially, 6 eight‐week‐old male mice (ICR, 18–22 g) were obtained from the Laboratory Animal Services Center, Nanjing University of Chinese Medicine. Animals were housed in individually ventilated cages (IVC) with up to five animals per cage, supplied with a standard diet and water without restriction. The mice were maintained under the following environment: 12‐h light/dark cycle (6: 00 a.m. to 6: 00 p.m.: lights on; 6: 00 p.m. to 6: 00 a.m. of next day: light off); temperature set at 23°C ± 2°C; humidity set at 55% ± 15%. The animal study was approved by the Animal Experimentation Ethics Committee of Nanjing University of Chinese Medicine (Reference No.: 202201A012) and guided by the Principles of Laboratory Animal Care (NIH publication No. 80‐23, revised 1996). We made every effort to minimize the suffering of animals used in the protocol. Mice were examined, handled every day, and weighed every week during treatment to ensure adequate health and suitability. The doses of tacrine administered to the mice were 24 mg/kg/day, dissolved in around 0.2 mL of sterile saline, respectively, via gavage. The mice were divided into two groups: an untreated control group and a tacrine group (24 mg/kg/day). The experiment lasted for 8 weeks, after which the mice were sacrificed by cervical dislocation. No anesthetic was used. The brain tissues of the mice were then prepared for the TdT‐mediated dUTP‐biotin nick end labeling (TUNEL) assay.

### 2.12 TUNEL Assay

To examine cell apoptosis, a TUNEL assay kit (Cat# C1098, Beyotime Biotechnology, Shanghai, China) was employed. The brain tissues were isolated and fixed in 4% paraformaldehyde solution, embedded in paraffin, and cut into 5 μm‐thick coronal plane sections. Then, the TUNEL assay kit was employed according to the manufacturer's instructions. Images were taken by an Axio Scan.Z1 digital slide scanner (Carl Zeiss, Thornwood, NY, USA).

### 2.13 Statistical Analysis

The statistical analyses were performed by using GraphPad Prism 10.0. No test for outliers was conducted. Inclusion and exclusion criteria were determined a priori. Exclusion criteria included any death during experimentation, and none met the exclusion criteria. All data were used for statistical analysis. The randomization method was used in the current research by using the “random number table”. In brief, six animals were weighed and numbered by weight from light to heavy and then allocated according to a “random number table”. The distribution of the data was tested with a Shapiro–Wilk normality test. All datasets passed the normality test. No formal a priori sample size calculation was performed. However, a pilot study involving five mice in each group was conducted prior to the main study. All of them exerted toxicity in the presence of brain apoptosis compared with the control group. No blinding was performed during data acquisition. Data are represented as Mean ± SEM. The significance of the difference was determined by one‐way ANOVA and Student's *t* test. The *p* < 0.05 was considered statistically significant.

## 3 Results

### 3.1 AChE Inhibitors Induce ER Stress in Neuronal Cells

**FIGURE 1:**
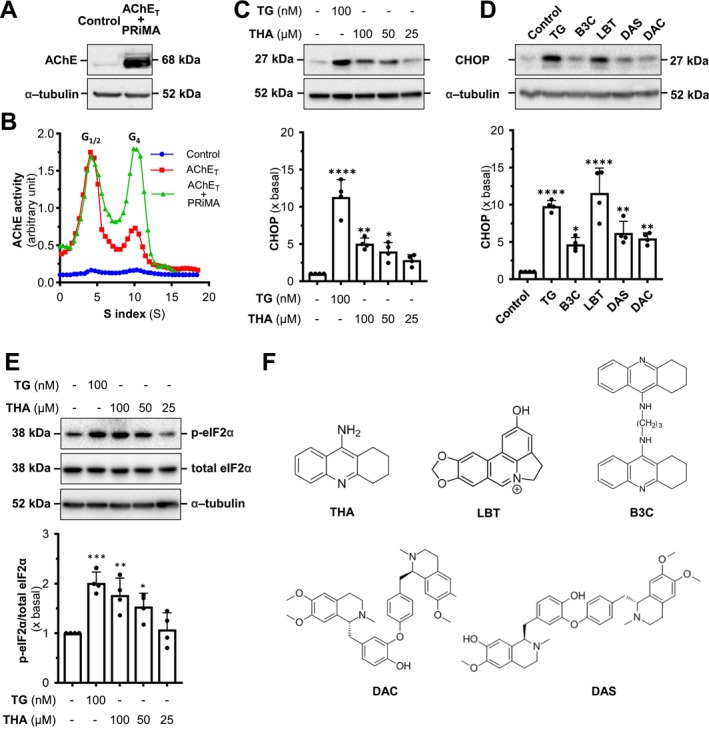
Tacrine and other AChE inhibitors induce ER stress in G4 AChE‐expressed cultured neuronal cells. (A) NG108‐15 cells were transfected with cDNAs encoding AChE_T_ and PRiMA. Protein lysates of the cultures were used for the Western blotting assay. (B) Total lysates from (A) were subjected to sucrose density gradient analysis. AChE activity was plotted as a function of the sedimentation value (S), estimated from the position of the sedimentation markers. Enzymatic activities are expressed as arbitrary units. (C) Cultured NG108‐15 cells, co‐transfected with AChE_T_ and PRiMA cDNAs, were exposed to tacrine (THA) at different doses for 24 h. The expression level of CHOP was determined (upper panel) and quantified (lower panel) from Western blotting. Thapsigargin (TG, 100 nM) served as a control. (D) AChE expressed cultured NG108‐15 cells, as in (C), were exposed to bis (3)‐cognitin (B3C; 20 μM), lycobetaine (LBT; 10 μM), daurisoline (DAS; 20 μM), and dauricine (DAC; 20 μM) for 24 h. The expression level of CHOP was determined (upper panel) and quantified (lower panel) from Western blotting. (E) AChE expressed cultured NG108‐15 cells, as in (C), were exposed to tacrine (THA) at different doses for 6 h. The expression levels of p‐eIF2α and total eIF2α were determined (upper panel) and quantified (lower panel) from Western blotting. (F) The chemical structures of tacrine (THA), lycobetaine (LBT), bis (3)‐cognitin (B3C), dauricine (DAC), and daurisoline (DAS). In (C) and (D), *α*‐tubulin served as an internal control. In (E), total eIF2α served as an internal control. Values are expressed as folds to basal (×basal), in mean ± SEM, *n* = 4 independent cell culture preparations. Statistical significance was analyzed by one‐way ANOVA followed by *Bonferroni post hoc test*, *p < 0.05; **p < 0.01; ***p < 0.001; ****p < 0.0001 vs. the control.

C/EBP homologous protein (CHOP) and eukaryotic initiation factor 2 alpha (eIF2α) are essential components of the UPR pathway, which is activated in response to ER stress. During ER stress, eIF2α is phosphorylated, resulting in a reduction in overall protein synthesis while promoting the translation of ATF4. The increased ATF4 subsequently triggers the expression of CHOP. CHOP is pivotal in mediating stress‐induced apoptosis and other cellular processes related to the stress response (Walter and Ron 2011; Hu et al. 2019). Thus, the expression levels of phosphorylated eIF2α (p‐eIF2α) and CHOP could serve as indicative markers to examine the effects of tacrine on ER stress. Here, the G4 AChE overexpressed cell model was established by transiently co‐transfecting with AChE_T_ and PRiMA. The total AChE protein in cultured NG108‐15 cells was upregulated by ~60‐fold after the transfection, indicating the success of DNA transfection (Figure 1A). The result of the sucrose density gradient verified the G4 AChE overexpression in the cultures (Figure 1B).

Thapsigargin, an ER stress inducer, served as a control to induce the stress (Figure 1C). Tacrine and other AChE inhibitors, including bis(3)‐cognitin (B3C), lycobetaine (LBT), daurisoline (DAS), and dauricine (DAC), were examined. The AChE overexpressed NG108‐15 cells were treated with or without thapsigargin, tacrine, or other AChE inhibitors for 24 h. The increase in CHOP expression, induced by tacrine, was in a dose‐dependent manner (Figure 1C). Tacrine at 100 μM induced a ~5‐fold increase in CHOP expression. Similarly, the tested AChE inhibitors induced the expression of CHOP at different levels (Figure 1D). Lycobetaine showed the strongest CHOP induction, exceeding a ~10‐fold increase at half of the concentration required by other inhibitors. In parallel, the expression level of p‐eIF2α was upregulated by tacrine in a dose‐dependent manner after 6 h of treatment (Figures 1E and S1), further verifying the induction of ER stress. The chemical structures of these inhibitors are shown (Figure 1F).

### 3.2 Trehalose Relieves the ER Stress Induced by Tacrine and Other AChE Inhibitors

**FIGURE 2:**
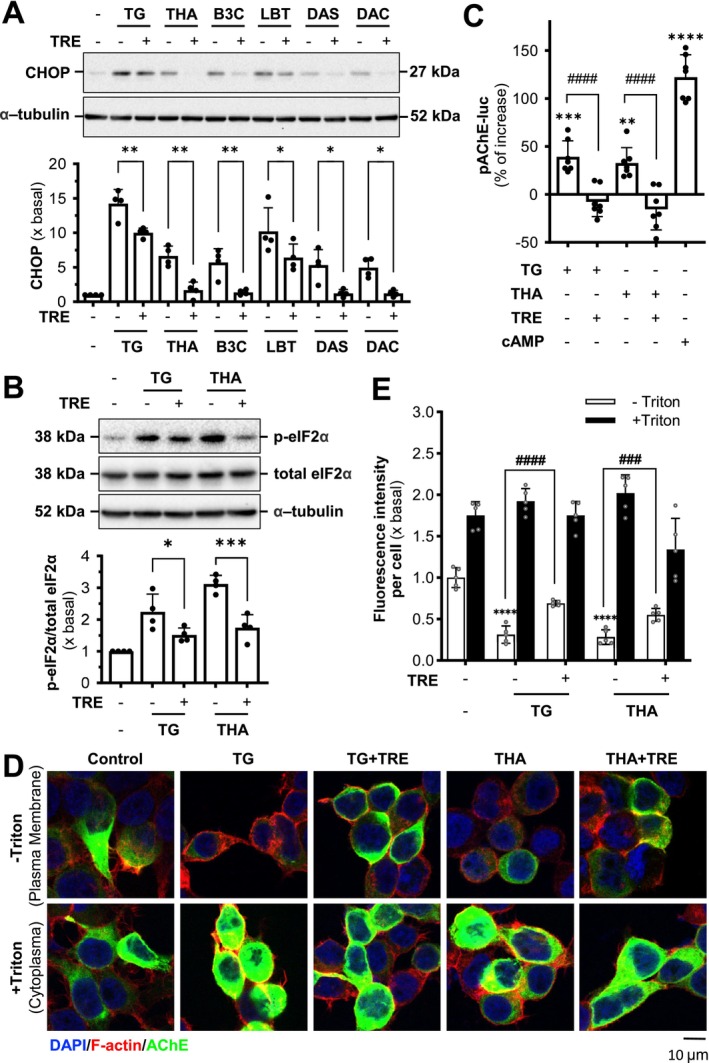
Trehalose relieves the tacrine‐induced ER stress in G4 AChE‐expressed cells. (A) Cultured NG108‐15 cells co‐transfected with AChE_T_ and PRiMA cDNAs were exposed to thapsigargin (TG, 100 nM), tacrine (THA, 100 μM), lycobetaine (LBT; 10 μM), daurisoline (DAS; 20 μM), dauricine (DAC; 20 μM), and bis(3)‐cognitin (B3C; 20 μM), with or without trehalose (TRE, 100 mM) for 24 h. The expression level of CHOP was determined (upper panel) and quantified (lower panel) from Western blotting. α‐Tubulin served as an internal control. (B) Cultured NG108‐15 cells co‐transfected with AChE_T_ and PRiMA cDNAs were treated with tacrine, with or without trehalose, for 6 h. The expression levels of p‐eIF2α and total eIF2α were determined (upper panel) and quantified (lower panel) from Western blotting. Total eIF2α served as an internal control. (C) Human *ACHE* promoter (pAChE‐Luc) cDNA was transiently transfected in NG108‐15 cells, then exposed to THA (100 μM) or TG (100 nM), with or without trehalose for 16 h. Bt_2_‐cAMP (dibutyryl‐cAMP, 1 mM) was used as a positive control. Luciferase assays were performed. (D) NG‐108‐15 cells, expressed with PRiMA‐linked G4 AChE, were seeded on coverslips at 10 × 10^4^ cells/mL. The cells were exposed to thapsigargin (TG; 100 nM) and tacrine (THA; 100 μM) with or without trehalose (TRE; 100 mM) for 24 h. Cells were then stained by anti‐AChE antibody with or without permeabilization with Triton X‐100 (0.2%). Nucleus (DAPI; blue), F‐actin (red), and AChE (green) are indicated. (E) Relative fluorescent intensity of AChE in each sample in panel (D). Values are expressed as a percentage of increase or folds to basal (×basal), in Mean ± SEM, *n* = 4–7 independent cell culture preparations. Statistical significance was analyzed by one‐way ANOVA followed by *Bonferroni post hoc test*, *p < 0.05; **p < 0.01; ***p < 0.001; ****p < 0.0001 vs. control or TRE‐treated; ^###^ *p* < 0.001; ^####^ *p* < 0.0001.

The CHOP and p‐eIF2α expressions were assessed by determining the magnitude of AChE inhibitor‐induced ER stress. The application of tacrine and the four AChE inhibitors upregulated the CHOP level over five‐fold in the AChE overexpressed NG108‐15 cells, as compared with the control group (Figure 2A). The co‐treatment of trehalose, a sugar able to stabilize folding proteins, significantly decreased the expression of CHOP, almost back to the control level, indicating that trehalose could reverse the AChE inhibitor‐ or thapsigargin‐induced ER stress. In addition, the upregulation of p‐eIF2α induced by tacrine could also be reversed by co‐treating with trehalose (Figure 2B). The applications of thapsigargin and tacrine have been shown to induce *AChE* promoter activity in a dose‐dependent manner (Liu et al. 2021). Here, this induction of *AChE* promoter was re‐confirmed by treatments with Bt_2_‐cAMP (dibutyryl‐cAMP), thapsigargin, and tacrine (Figure 2C). As expected, the co‐treatment with trehalose suppressed the promoter induction, suggesting that trehalose could play a role in maintaining cellular homeostasis under stress.

The immunofluorescence staining of intracellular AChE was employed to visualize the effect of tacrine on AChE expression and its intracellular location. After the overexpression of AChE, the NG‐108‐15 cells were exposed to thapsigargin and tacrine, with or without trehalose, for 24 h. The cells were stained with anti‐AChE antibody, DAPI, rhodamine phalloidin, with or without Triton X‐100. DAPI stained the nucleus, and rhodamine phalloidin stained the cytoskeleton. In the presence of Triton X‐100, the intracellular AChE was detected (Figure 2D). A slight upregulation of intracellular AChE level was detected in the groups of thapsigargin and tacrine. Without Triton X‐100, the staining of AChE was mostly revealed on the cell surface. In the control group, AChE staining was detected at a relatively high level on the surface, indicating that AChE was anchored to the PM. In the tacrine‐treated group, the intensity of the membrane enzyme was significantly decreased, indicating that AChE might not be transported to the cell surface (Figure 2D,E). In the groups of co‐treatment with trehalose, the intensities were significantly increased, indicating that trehalose could reverse the ER stress, as induced by tacrine, and which assisted the assembly and transport of AChE to the PM. Thapsigargin served as a positive control.

**FIGURE 3:**
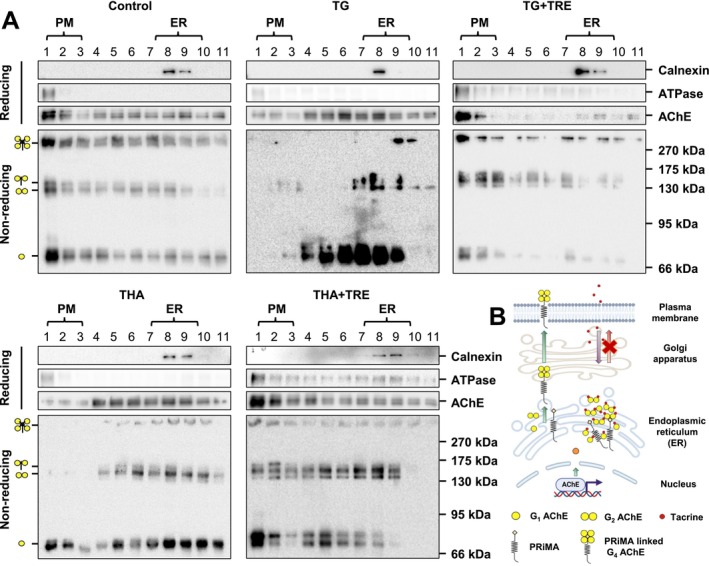
Trehalose reverses the tacrine‐induced AChE accumulation in the ER. (A) Cultured NG108‐15 cells co‐transfected with cDNAs encoding AChE_T_ and PRiMA were exposed to thapsigargin (TG, 100 nM) or tacrine (THA, 100 μM) with or without trehalose (TRE, 100 mM) for 24 h. The cell lysates underwent subcellular fractionation. The distribution of proteins was determined by Western Blotting and then analyzed by corresponding antibodies. Representative gel is shown, *n* = 4 independent cell culture preparations. (B) Schematic diagram of tacrine disrupting the proper assembly of AChE in ER and leading to ER stress.

Based on the results of immunofluorescence staining, we hypothesize that the tacrine‐induced ER stress could be a result of disrupting the assembly of AChE oligomers, whereas trehalose may alleviate this ER stress by facilitating proper enzyme assembly. To investigate how tacrine and trehalose influence the assembly and transport of AChE, the protein extracts from the total lysate and ER fraction of AChE overexpressed in NG108‐15 cells were subjected to reducing and non‐reducing SDS gel. The ER fraction showed an increased amount of G2 and G4 forms of AChE (Figure S2). In addition, the subcellular fractionation using linear iodixanol gradients was performed to visualize the changes in the distribution of different AChE isoforms at the subcellular level. On top of the gradient, the PM fraction was enriched, indicated by the PM marker (Na^+^/K^+^‐ATPase), whereas the ER fraction was enriched at the bottom of the gradient, indicated by the ER marker (calnexin) (Figure 3A,B). In the control group, AChE exhibited the classic transport from the ER to the PM. After 24 h of exposure to thapsigargin or tacrine, an increase in total AChE within the ER fraction and a decrease in the PM fraction were identified in the reducing gel electrophoresis. Meanwhile, the non‐reducing gel electrophoresis was employed to further verify the AChE isoforms distribution in different cellular fractions (Figure 3A). With the treatment of thapsigargin or tacrine, the amounts of G1 to G4 AChE were highly accumulated in the ER fraction, whereas the membrane‐bound G4 AChE was significantly decreased (Figure 3B). The co‐treatment of trehalose significantly restored the stress caused by thapsigargin or tacrine, that is, increased membrane‐bound AChE but decreased the enzyme in the ER. The results were consistent with the results in immunofluorescence staining.

### 3.3 Tacrine Induces ER Stress in Cells Expressing Different Forms of AChE and BChE

**FIGURE 4:**
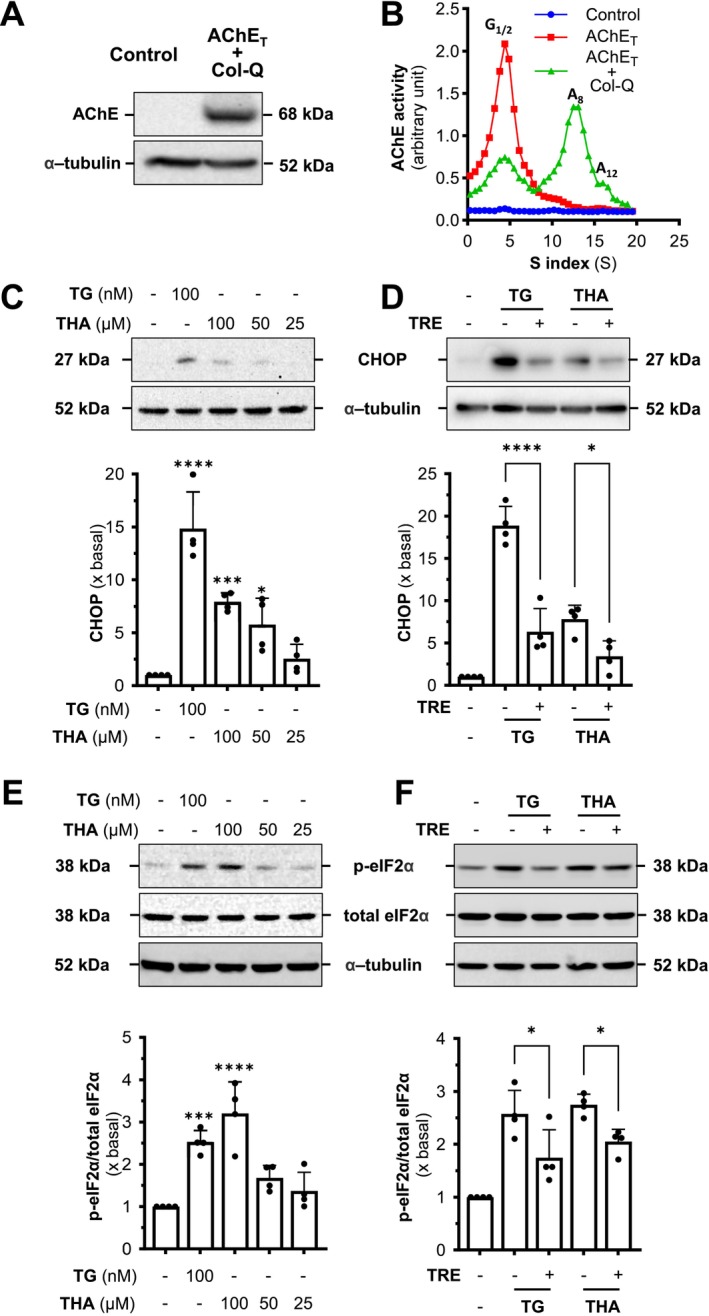
Trehalose relieves the tacrine‐induced ER stress in asymmetric AChE‐expressed cells. (A) HEK293T cells were transfected with or without AChE_T_ and ColQ cDNAs. Protein lysates of the cultures were used for Western blotting assay. (B) Total lysates from HEK293T cells expressed asymmetric AChE, as in (A), were subjected to sucrose density gradient analysis. AChE activity was plotted as a function of the sedimentation value (S), estimated from the position of the sedimentation markers. Data were expressed as arbitrary units. (C) Cultured HEK293T cells expressing asymmetric AChE were exposed to tacrine (THA) at different doses for 24 h. Thapsigargin (TG, 100 nM) served as a control. The expression level of CHOP was determined (upper panel) and quantified (lower panel) using Western blotting. α‐Tubulin served as an internal control. (D) Treatment of tacrine (THA, 100 μM) as in (C) with or without trehalose (TRE, 100 mM) for 24 h. The expression level of CHOP was determined (upper panel) and quantified (lower panel) using Western blotting. (E) Cultured HEK293T cells expressing asymmetric AChE were treated with different levels of tacrine for 4 h. The expression levels of p‐eIF2α and total eIF2α were determined (upper panel) and quantified (lower panel) from Western blotting. Total eIF2α served as an internal control. (F) Cultured HEK293T cells expressing asymmetric AChE were treated with thapsigargin (TG, 100 nM) or tacrine (THA, 100 μM) with or without trehalose (TRE, 100 mM) for 4 h. The expression level of p‐eIF2α was determined (upper panel) and quantified (lower panel) from Western blotting. Values are expressed as folds to basal (×basal), in Mean ± SEM, *n* = 4 independent cell culture preparations. Statistical significance was analyzed by one‐way ANOVA followed by *Bonferroni post hoc test*, *p < 0.05; ***p < 0.001; ****p < 0.0001 vs. control or TRE‐treated.

Asymmetric AChE, specifically isoforms A8 and A12, is derived from tetrameric AChE linked to the ColQ subunit, which anchors to the basal lamina at vertebrate neuromuscular junctions (Massoulié et al. 1998a). The catalytic subunit association is mainly based on a tight interaction between the four C‐terminal t‐peptides of AChE_T_ subunits and a proline‐rich attachment domain in ColQ (Massoulié and Millard 2009). Here, tacrine was tested for its induction of ER stress in cells producing asymmetric AChE. By transiently co‐transfecting AChE_T_ and ColQ in HEK293T cells (Figure 4A), the forms of A8 and A12 AChE were identified (Figure 4B). In the ColQ‐associated AChE overexpressed cells, tacrine was able to induce ER stress, as evidenced by the upregulation of CHOP in a dose‐dependent manner (Figure 4C). Furthermore, the co‐treatment of trehalose demonstrated the alleviation of the tacrine‐induced ER stress in the cultures (Figure 4D). The induction of p‐eIF2α expression showed a similar trend after 4 h treatment (Figure 4E), as well as the reversal of the stress by trehalose application (Figure 4F).

**FIGURE 5:**
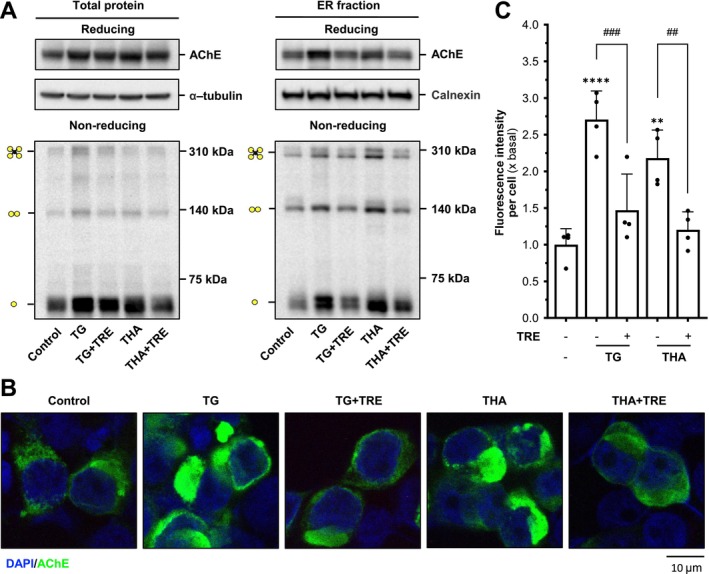
Trehalose restores the proper assembly of asymmetric AChE in ER stress cells. (A) Cultured HEK293T cells co‐transfected with AChE_T_ and ColQ cDNAs were exposed to thapsigargin (TG, 100 nM) and tacrine (THA, 100 μM) with or without trehalose (TRE, 100 mM) for 24 h. Total lysates and ER fraction were analyzed by Western blotting with reduced and non‐reduced gels. (B) HEK293T cells, expressing asymmetric AChE, were seeded on coverslips at 10 × 10^4^ cells/mL. The cells were exposed to drugs as in (A). Cells were then stained with anti‐AChE antibody with permeabilization of Triton X‐100 (0.2%). Nucleus (DAPI; blue), F‐actin (red), and AChE (green) are indicated. (C) Relative fluorescent intensity of AChE in each sample in panel (B). Values are expressed as folds to basal (×basal), in Mean ± SEM, *n* = 4 independent cell culture preparations. Statistical significance was analyzed by one‐way ANOVA followed by *Bonferroni post hoc test*, **p < 0.01; ****p < 0.0001 vs. the control; ^##^ *p* < 0.01; ^###^ *p* < 0.001.

To investigate AChE assembly within the ER, we extracted the ER fraction and performed non‐reducing gel electrophoresis. In addition to a slight increase in total AChE protein, the groups exposed to thapsigargin and tacrine exhibited markedly higher AChE accumulation in the ER fraction, including G1, G2, and G4 forms, whereas the co‐treatment with trehalose significantly reduced this accumulation (Figure 5A). These findings suggest that tacrine substantially disrupts the assembly of ColQ‐associated AChE. Immunofluorescence staining of AChE was employed to visualize the effect of tacrine on ColQ‐associated AChE expression and localization, in the presence of Triton X‐100 (Figure 5B). The intracellular AChE was visualized and quantified by the fluorescence signal intensity: the levels of AChE in thapsigargin and tacrine‐exposed groups showed a significant increase (Figure 5C). In contrast, the co‐treatment of trehalose showed a significant decrease, indicating that thapsigargin and tacrine led to the accumulation of ColQ‐associated AChE in ER fraction, whereas trehalose could reverse the accumulation, similar to the PRiMA‐linked G4 enzyme.

**FIGURE 6:**
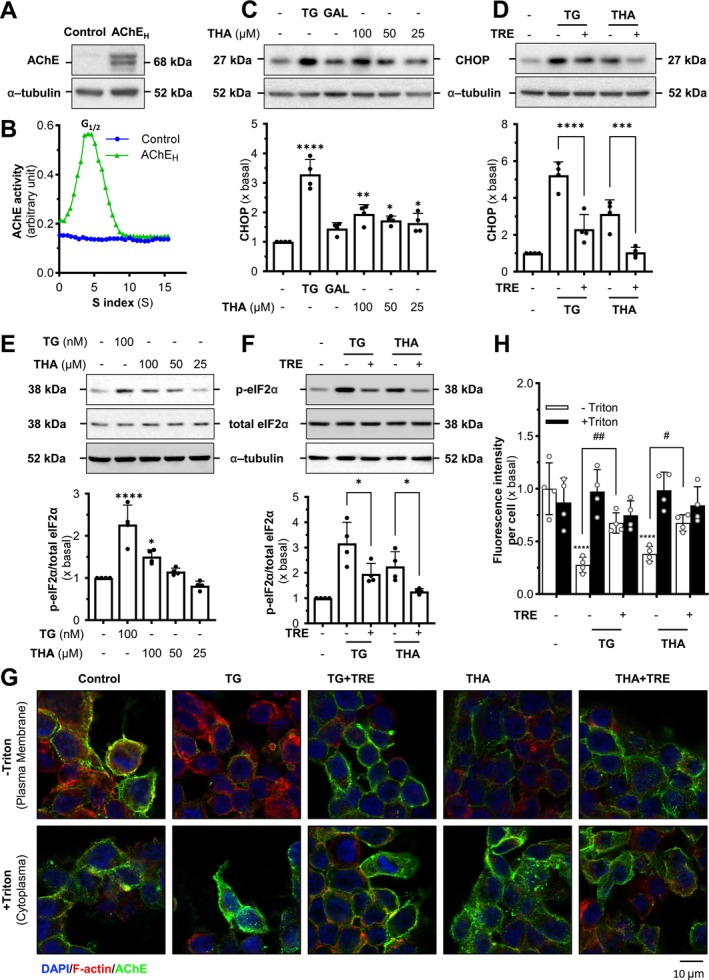
Trehalose relieves the tacrine‐induced ER stress in G2a AChE‐expressed cells. (A) HEK293T cells were transfected with or without AChE_H_ cDNAs. Protein lysates of the cultures were used for Western blotting assay. (B) Total lysates from HEK293T cells expressing GPI‐linked G2a AChE, as in (A), were subjected to sucrose density gradient analysis. AChE activity was plotted as a function of the sedimentation value (S), estimated from the position of the sedimentation markers. Data were expressed as arbitrary units. (C) Cultured HEK293T cells expressing G2a AChE were exposed to tacrine (THA) at different doses, and galantamine (GAL, 10 μM) for 24 h. Thapsigargin (TG, 100 nM) served as a control. The expression level of CHOP was determined (upper panel) and quantified (lower panel) using Western blotting. α‐tubulin served as an internal control. (D) Treatment of tacrine (THA, 100 μM) as in (C) with or without trehalose (TRE, 100 mM) for 24 h. The expression level of CHOP was determined (upper panel) and quantified (lower panel) using Western blotting. (E) Cultured HEK293T cells expressing G2a AChE were treated with different levels of tacrine for 4 h. The expression levels of p‐eIF2α and total eIF2α were determined (upper panel) and quantified (lower panel) from Western blotting. Total eIF2α served as an internal control. (F) Cultured HEK293T cells expressing G2a AChE were treated with thapsigargin (TG, 100 nM) or tacrine (THA, 100 μM) with or without trehalose (TRE, 100 mM) for 4 h. The expression levels of p‐eIF2α and total eIF2α were determined (upper panel) and quantified (lower panel) from Western blotting. (G) HEK293T cells, expressed with G2a AChE, were seeded on coverslips at 10 × 10^4^ cells/mL. The cells were exposed to thapsigargin (TG; 100 nM) and tacrine (THA; 100 μM) with or without trehalose (TRE; 100 mM) for 24 h. Cells were then stained by anti‐AChE antibody with or without permeabilization with Triton X‐100 (0.2%). Nucleus (DAPI; blue), F‐actin (red), and AChE (green) are indicated. (H) Relative fluorescence intensity of AChE in each sample in panel (G). Values are expressed as folds to basal (×basal), in Mean ± SEM, *n* = 4 independent cell culture preparations. Statistical significance was analyzed by one‐way ANOVA followed by *Bonferroni post hoc test*, *p < 0.05; **p < 0.01; ***p < 0.001; ****p < 0.0001 vs. control or TRE‐treated; ^#^ *p* < 0.01; ^##^ *p* < 0.001.

The dimeric form of AChE (G2a) is formed by AChE_H_ in red blood cells and is characterized by its C‐terminal peptide containing one or two cysteines near the catalytic domain, along with a signal for the addition of a glycophosphatidylinositol (GPI) anchor. Here, the G2a AChE overexpressed cells were obtained by transiently transfecting with AChE_H_ in cultured HEK293T cells. The induced expression of AChE protein, as well as the G2a form in sucrose density gradient, was identified (Figure 6A,B). The exposures of thapsigargin and tacrine significantly induced the upregulation of CHOP (Figure 6C), indicating the induction of ER stress. In the meantime, trehalose reduced the upregulation of CHOP, as induced by thapsigargin or tacrine (Figure 6D). In addition, the level of p‐eIF2α was also induced by tacrine in the AChE_H_‐expressed cells (Figure 6E), and the induction was reversed by trehalose (Figure 6F).

Immunostaining was performed to further visualize the distribution of intracellular G2a AChE. The cells were stained with the antibody against AChE, with or without permeabilization by Triton X‐100 (Figure 6G,H). In the control group, the fluorescence intensities with or without Triton X‐100 were similar. In the group of thapsigargin and tacrine, the fluorescence intensity was significantly lower in the stained cells without Triton X‐100, indicating a decrease in AChE expression level on the PM. In comparison, the co‐treatment of trehalose could rescue the decrease in AChE expression level on the PM. These results indicate that the G2a AChE assembly and transportation could be disrupted by tacrine and accumulate in the ER. At the same time, trehalose, as a chemical chaperone, could help the proper assembly of G2a AChE and reduce ER stress.

**FIGURE 7:**
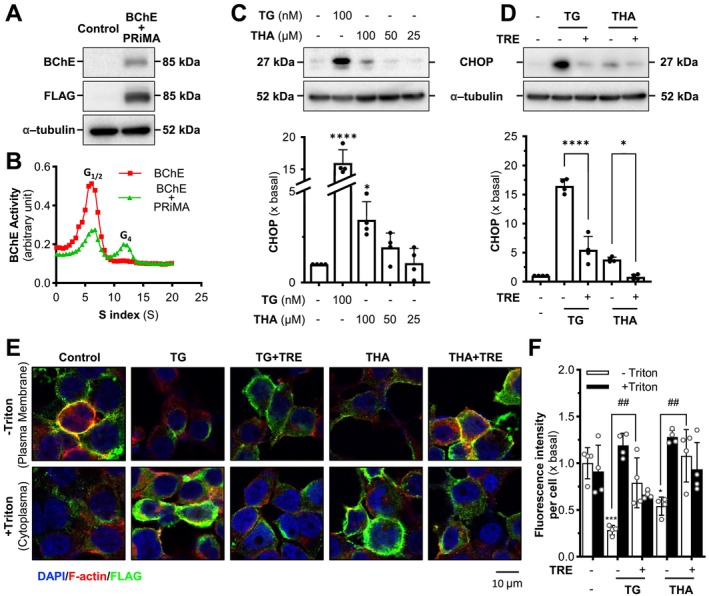
Trehalose relieves the tacrine‐induced ER stress BChE‐expressed cells. (A) HEK293T cells were transfected with cDNAs encoding BChE_T_ tagged with a FLAG epitope at the N terminus and PRiMA. Protein lysates of the cultures were analyzed for Western blotting, recognized by anti‐BChE and anti‐FLAG antibodies. (B) The lysates from BChE‐expressed HEK293T cells were subjected to sucrose density gradient analysis, as in Figure 1. Data were expressed as arbitrary units. (C) BChE‐expressed HEK293T cells were exposed to tacrine (THA) at different doses for 24 h. Thapsigargin (TG, 100 nM) served as a control. The expression level of CHOP was determined (upper panel) and quantified (lower panel) using Western blotting. α‐Tubulin served as an internal control. (D) BChE expressed HEK293T cells were exposed to thapsigargin (TG, 100 nM) and tacrine (THA, 100 μM) with or without trehalose (TRE, 100 mM) for 24 h. The expression level of CHOP was determined (upper panel) and quantified (lower panel) using Western blotting. (E) BChE‐expressed HEK293T cells were seeded on coverslips in 10 × 10^4^ cells/mL. The cells were exposed to drugs as in (D). Cells were then stained by anti‐FLAG antibody with or without permeabilization with Triton X‐100. Nucleus (DAPI; blue), F‐actin (red), and FLAG (green) are indicated (right panel). (F) Quantification of fluorescence intensity in (E) was shown. Values are expressed as folds to basal (×basal), in Mean ± SEM, *n* = 4 independent cell culture preparations. Statistical significance was analyzed by one‐way ANOVA followed by *Bonferroni post hoc test*, *p < 0.05; ***p < 0.001; ****p < 0.0001 vs. control or TRE‐treated; ^##^ *p* < 0.001.

BChE, the other cholinesterase in vertebrates, could hydrolyze ACh. BChE could also be linked with PRiMA and anchored to the cell surface in neuronal cells, similar to that of G4 AChE (Chatonnet and Lockridge 1989). Tacrine inhibits the activity of BChE, indicating that it has the potential to disrupt the assembly of G4 BChE intracellularly, leading to ER stress. We co‐transfected BChE cDNA tagged with a FLAG epitope at the N terminus and PRiMA cDNA in cultured HEK293T cells. The overexpression of the BChE catalytic band at ~85 kDa was over 20‐fold, as recognized by anti‐BChE or anti‐FLAG antibodies (Figure 7A). The expressed forms were G1/2 and G4, as expected (Figure 7B). The CHOP expression was increased by tacrine in a dose‐dependent manner: the maximal induction at 100 mM tacrine was at ~5‐fold (Figure 7C), and the induction could be reversed by the co‐treatment of trehalose (Figure 7D). In immunofluorescence staining, the cells were stained with anti‐FLAG antibody, DAPI, and rhodamine phalloidin, with or without Triton X‐100. In the presence of Triton X‐100, the staining of FLAG revealed the level of intracellular BChE (Figure 7E), which was increased slightly in treatments of thapsigargin and tacrine. Without Triton X‐100, the staining of FLAG was revealed on the cell surface. In thapsigargin and tacrine groups, the FLAG level was significantly decreased, indicating that BChE was not able to transport to the cell surface properly (Figure 7E,F). In the group of co‐treatment with trehalose, the FLAG‐tagged BChE was significantly increased, indicating that the trehalose could reverse the ER stress, as induced by tacrine, which assisted the transport of G4 BChE to the membrane (Figure 7E,F).

### 3.4 Tacrine Induces ER Stress and Leads to Apoptosis In Vivo

**FIGURE 8:**
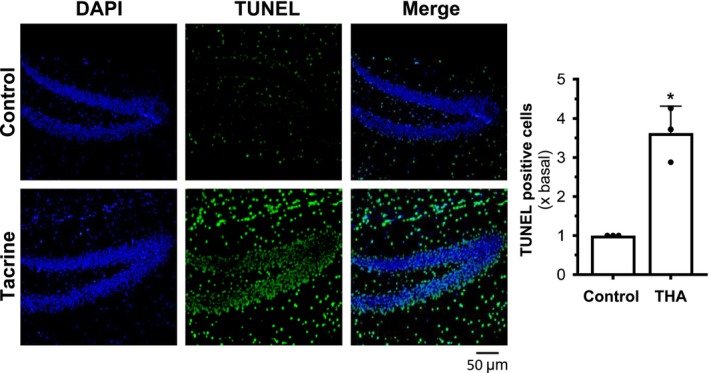
AChE inhibitors induce apoptosis in the mouse brain. The dose of tacrine administered to mice was 24 mg/kg/day by gavage. Mice were treated for 8 weeks, and then the brain tissue was examined with the TUNEL assay (left panel). Quantification of TUNEL‐positive cells was shown (right panel). Values are expressed as folds to basal (×basal), in Mean ± SEM, *n* = 3 mice. Statistical significance was analyzed by unpaired two‐tailed Welch's *t*‐test, *p < 0.05.

On the basis of the above in vitro results, we have a hypothesis that tacrine was likely to induce the ER stress in vivo. To further investigate the effect of tacrine on ER stress in vivo, we conducted TUNEL assays in tacrine‐treated mice. The mice received oral administration of a high dose of tacrine for a duration of 8 weeks; then, the brain tissues were collected for analysis. The TUNEL assay was performed to assess the level of apoptosis in the tissue. The results revealed a significantly higher level of apoptosis in the tacrine‐exposed group, as compared to control (Figure 8). These findings support the hypothesis that tacrine intake could induce ER stress in mice that express AChE or even BChE.

## 4 Discussion

The hepatotoxicity of tacrine has been well‐documented, and it was believed to be the main reason for its adverse effects and withdrawal from the market (Watkins et al. 1994). Besides, tacrine and other AChE inhibitors, but not all, could induce ER stress in cells expressing AChE (Liu et al. 2021). The accumulation and deposition of the ER stress‐induced misfolded proteins could lead to neurodegenerative diseases, such as cerebral ischemia, sleep apnoea, Alzheimer's disease, multiple sclerosis, amyotrophic lateral sclerosis, prion diseases, and familial encephalopathy having neuroserpin inclusion bodies. The stress could disrupt various cell signaling pathways, impair neuronal connectivity, and ultimately lead to cell death (Soto 2003; Lindholm et al. 2006). Here, we hypothesize that the ER stress caused by tacrine, as well as other AChE inhibitors, could be because of the improper assembly and transport of oligomeric AChE in the ER and finally contributes to the adverse effects.

Here, we first verified that tacrine and other AChE inhibitors could induce ER stress in AChE/BChE overexpressed cells. Trehalose, one of the known ER stress reducers, could reverse the tacrine‐induced ER stress. The stress could be at three levels: (i) an upregulation of AChE transcription; (ii) the assembly of oligomeric AChE; and (iii) the transport from ER to PM. The increased AChE expression could be an outcome of the stress‐induced UPR, for example, cAMP signaling, which has been known to induce gene transcription or epigenetic regulation (Liu et al. 2021). The stress‐induced AChE expression could be just one of the induced responses. Using subcellular fractionation and non‐reducing gel electrophoresis of G4 form AChE, the G1 and G2 forms were significantly accumulated in the ER under the treatment of tacrine. In parallel, the G4 form was reduced in the ER, suggesting the assembly of tetrameric AChE was defective. In parallel, a defect in the transport of AChE from the ER to the PM was identified. This transportation problem could be the result of improper folding of tetrameric AChE. Besides the G4 form of AChE, the ColQ‐linked AChE, the GPI‐linked AChE, and the PRiMA‐linked BChE were also affected similarly by the tacrine‐induced stress. The rationale could be very similar to G4 AChE that the improper assembly was the cause of the protein transport defect. Thus, the AChE inhibitor‐induced aggregation of improperly assembled AChE and BChE could induce ER stress, which thereafter leads to cell apoptosis. However, how different isoforms of AChE were assembled and transported from the ER to the PM under the effect of tacrine still remains unclear.

Not all AChE inhibitors can induce ER stress (Liu et al. 2021). To gain an understanding of how specific AChE inhibitors induce ER stress, the three‐dimensional structure of AChE and its complexes with the inhibitors could be an answer for the discrepancy. The X‐ray crystallographic studies have revealed that AChE has a deep and narrow gorge, penetrating halfway into the enzyme. This cavity, known as the “active site gorge”, containing the AChE catalytic triad, is coated with aromatic side chains. The alignment of aromatic side chains in the gorge is a highly conserved three‐dimensional structure across evolution (Sussman et al. 1991; Wiesner et al. 2007). In the tacrine‐AChE complex, the residues of F330 and W279 show different positioning. The tacrine moiety is stacked against W84, and its ring nitrogen forms a hydrogen bond with the main‐chain carbonyl oxygen of H440 (Sussman et al. 1993). This interaction was further validated by photoaffinity labelling by the photosensitive probe 3H‐labeled p‐(N,N‐dimethylamino) benzenediazonium fluoroborate (Harel et al. 1993). Additionally, the molecular dynamic simulations of the AChE dimer complexed with tacrine in solution revealed fluctuations in the width of the active site, allowing the substrate entry to the active site via the side walls of the gorge (Wlodek et al. 1997; Xu et al. 2017). These findings provide a possible structural explanation of how the tacrine could affect the oligomerization and trafficking of AChE in the ER; however, the interactions of other inhibitors with AChE complex are not resolved.

Molecular chaperones play a crucial role in maintaining proteostasis by facilitating protein folding and reducing proteotoxicity, which generally assists proper protein folding under both physiological and stress conditions (Hartl 1996). In addition to their primary function in protein folding, chaperones are involved in assembling and disassembling protein complexes, presenting protein substrates for degradation, and preventing protein aggregation (Wright et al. 2015). Trehalose has been identified as a chemical chaperone to enhance protein folding and stability. Trehalose has two glucose units connected via a strong α‐1,1 bond, making it stable in acidic and high‐temperature conditions and applicable as a useful agent in biological preservation. Early work indicates that trehalose is a more stable and versatile protectant than traditional protein chaperones, effectively preventing the aggregation of cytoplasmic and membrane proteins (Liang et al. 2012). Here, trehalose shows robust effects in relieving tacrine‐ and AChE inhibitor‐induced ER stresses, which significantly reduces the accumulation of G1 and G2 AChE in the ER and restores the AChE oligomer to assemble properly and transport to the PM.

AChE inhibitors are being used as drugs to treat AD with daily intake. Many of them have different levels of adverse effects, even fatal. The ER stress, induced by tacrine and AChE inhibitors, may have potential adverse effects on the human body. However, the chemical chaperone offers us a method where the AChE inhibitors could be taken together with adjuvants, for example, trehalose, which helps protein folding and reduces the ER stress. Besides, AChE inhibitors could be considered to take a lower dose during the treatment, to avoid the induced ER stress. In this way, the ER stress could be relieved, and the adverse effects are prevented. However, not all AChE inhibitors are capable of inducing ER stress and causing such adverse effects (Liu et al. 2021). To further investigate why some AChE inhibitors could disrupt the assembly of AChE, the molecular structure of AChE inhibitors and AChE molecules should be learned by molecular docking, X‐ray crystallographic studies and/or cryo‐EM. With a large amount of such analysis, the characteristics of those AChE inhibitors disrupting the AChE assembly could be learned by machine learning. Therefore, this could serve as one of the criteria to screen new potential AChE inhibitors for the treatment of AD, in order to reduce the potential adverse effects. It could also be utilized to design the new multifunctional analogues and hybrids of tacrine or other AChE inhibitors so that these candidates could have less adverse effects and be used to treat AD.

## Author Contributions

**Xiaoyang Wang:** conceptualization, formal analysis, investigation, methodology, validation, visualization, writing – original draft, writing – review and editing. **Yingjie Xia:** investigation, methodology, validation. **Maggie Suisui Guo:** investigation, validation. **Jiahui Wu:** investigation, methodology. **Ajiaikebaier Dilidaer:** investigation, validation. **Jin Gao:** conceptualization, investigation. **Tina Tingxia Dong:** resources, supervision, funding acquisition. **Yue Zhu:** investigation, methodology. **Karl Wah Keung Tsim:** conceptualization, supervision, visualization, methodology, funding acquisition, project administration, writing – review and editing.

## Conflicts of Interest

The authors declare no conflicts of interest.

## Peer Review

The peer review history for this article is available at https://www.webofscience.com/api/gateway/wos/peer‐review/10.1111/jnc.70178.

## Supporting information


**Figure S1:** Tacrine induces the phosphorylation of eIF2α at different time points. Cultured NG108‐15 cells co‐transfected with AChET and PRiMA cDNAs were treated with tacrine, or thapsigargin, for 0.5 to 24 h. The expression level of p‐eIF2α was determined by Western blotting. Total eIF2α served as an internal control. Representative gel is shown, *n* = 4 independent cell culture preparations.
**Figure S2:** Tacrine induces improper assembly and accumulation of AChE in ER. Cultured NG108‐15 cells co‐transfected with AChE_T_ and PRiMA cDNAs were exposed to thapsigargin (TG, 100 nM) and tacrine (THA, 100 μM) with or without trehalose (TRE, 100 mM) for 24 h. Total lysates and ER fraction lysates were analyzed by Western blotting. Representative gel is shown, *n* = 4 independent cell culture preparations.
**Table S1:** Full statistical report.

## Data Availability

The data that support the findings of this study are available from the corresponding author upon reasonable request.
