# Supplementary material for: The Tacrine‐Induced Endoplasmic Reticulum Stress in AChE‐Expressed Cells Leads to Improper Assembly and Transport of the Oligomeric Enzyme: Reversal by Trehalose
Source: J Neurochem. 2025 Aug 5;169(8):e70178. doi: 10.1111/jnc.70178 (PMC12322807; doi:10.1111/jnc.70178)
Supplement: Supplementary file 1 — Figure S1: Tacrine induces the phosphorylation of eIF2α at different time points. Cultured NG108‐15 cells co‐transfected with AChET and PRiMA cDNAs were treated with tacrine, or thapsigargin, for 0.5 to 24 h. The expression level of p‐eIF2α was determined by Western blotting. Total eIF2α served as an internal control. Representative gel is shown, n = 4 independent cell culture preparations. Figure S2: Tacrine induces improper assembly and accumulation of AChE in ER. Cultured NG108‐15 cells co‐transfected with AChET and PRiMA cDNAs were exposed to thapsigargin (TG, 100 nM) and tacrine (THA, 100 μM) with or without trehalose (TRE, 100 mM) for 24 h. Total lysates and ER fraction lysates were analyzed by Western blotting. Representative gel is shown, n = 4 independent cell culture preparations. Table S1: Full statistical report. [file JNC-169-0-s001.pdf]

The tacrine-induced endoplasmic reticulum stress in AChE-expressed cells leads to improper assembly and transport of the oligomeric enzyme: reversal by trehalose

Xiaoyang WANG[1], Yingjie XIA[1,2], Maggie Suisui GUO[1], Jiahui WU[1,2], Ajiaikebaier DILIDAER[1], Jin GAO[1,3], Tina Tingxia DONG[1,2], Yue ZHU[4], Karl Wah Keung TSIM [1,2*]

[1]Division of Life Science, Center for Chinese Medicine and State Key Laboratory of Molecular Neuroscience, The Hong Kong University of Science and Technology, Hong Kong, China

[2]Shenzhen Key Laboratory of Edible and Medicinal Bioresources, The Hong Kong University of Science and Technology, Shenzhen, China

[3]Department of Neurobiology and Cellular Biology, Xuzhou Medical University, Xuzhou, Jiangsu, China

[4]Jiangsu Key Laboratory for High Technology Research of TCM Formulae and Jiangsu Collaborative Innovation Center of Chinese Medicinal Resources Industrialization, Nanjing University of Chinese Medicine, Nanjing, Jiangsu, China

*Correspondence should be addressed to: Professor Karl W. K. Tsim, Shenzhen Key Laboratory of Edible and Medicinal Bioresources, Shenzhen Research Institute, Shenzhen, 518000, China; e-mail: botsim@ust.hk

Supplementary_Figure S1

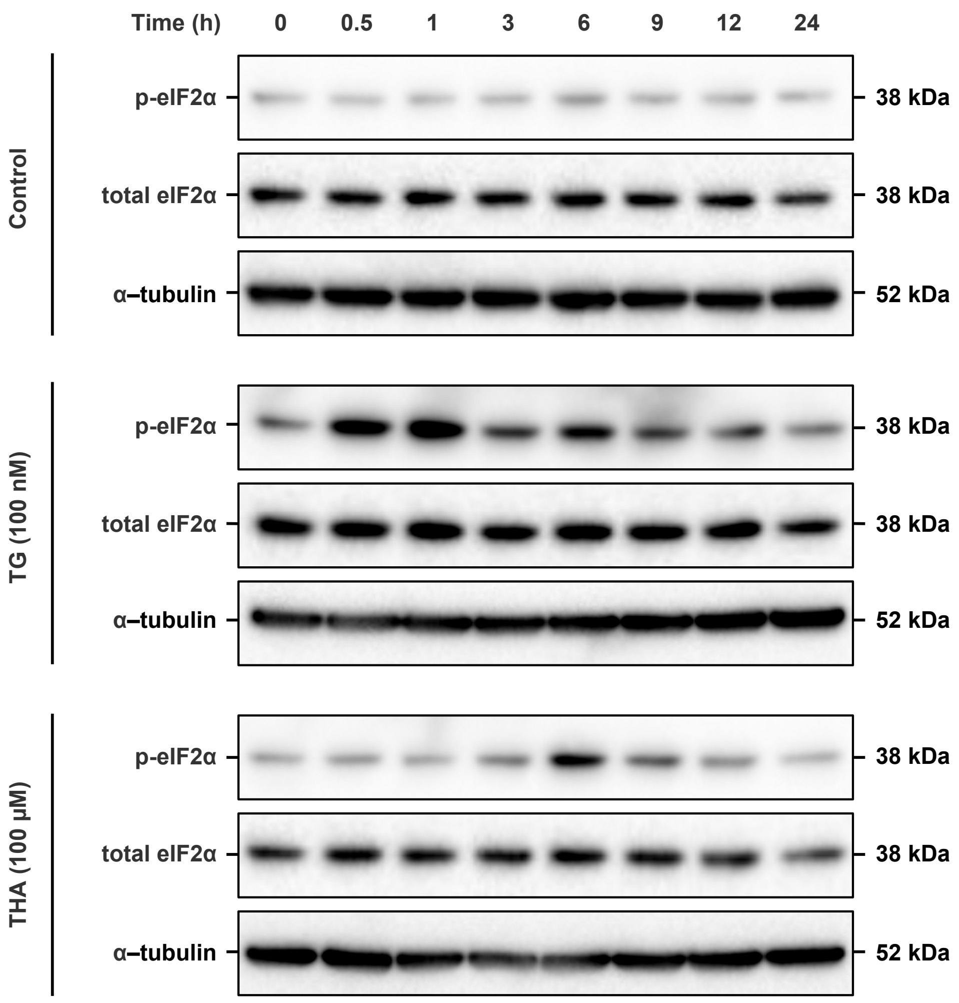

Figure S1. Tacrine induces the phosphorylation of eIF2α at different time points.

Cultured NG108-15 cells co-transfected with $AChE_T$ and PRiMA cDNAs were treated with tacrine, or thapsigargin, for 0.5 to 24 hours. The expression level of p-eIF2α was determined by Western blotting. Total eIF2α served as an internal control. Representative gel is shown, *n* = 4 independent cell culture preparations.

Supplementary_Figure S2

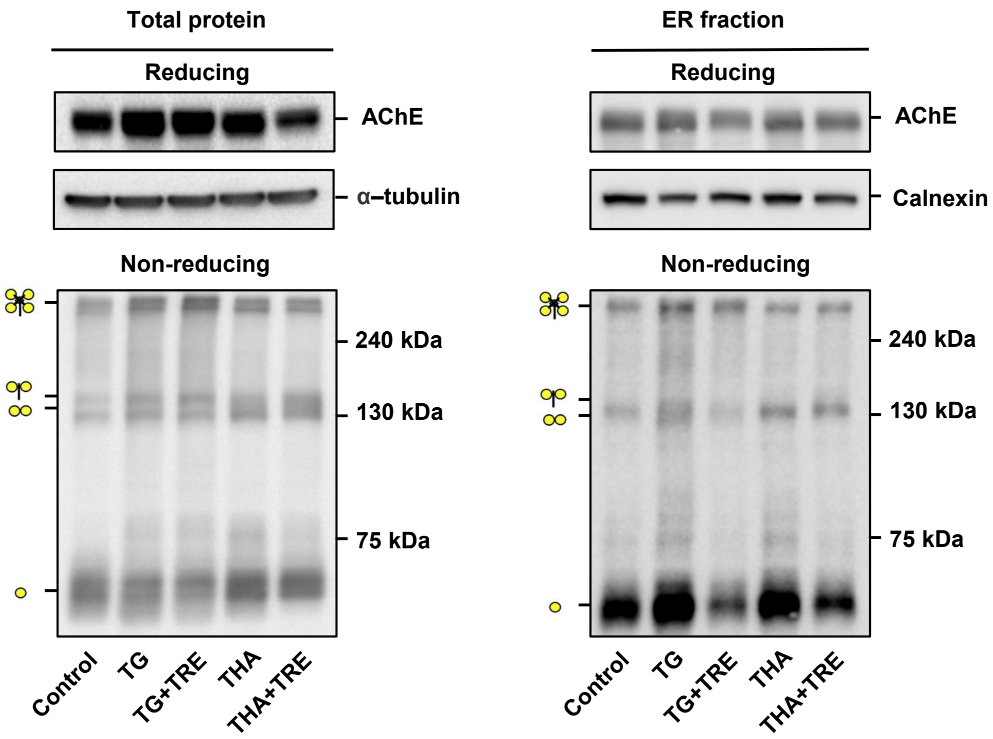

Figure S2. Tacrine induces improper assembly and accumulation of AChE in ER.

Cultured NG108-15 cells co-transfected with $AChE_T$ and PRiMA cDNAs were exposed to thapsigargin (TG, 100 nM) and tacrine (THA, 100 μM) with or without trehalose (TRE, 100 mM) for 24 hours. Total lysates and ER fraction lysates were analysed by Western blotting. Representative gel is shown, *n* = 4 independent cell culture preparations.

Supplementary_Table_1 Full statistical report

Fig. 1C	ANOVA table	F (DFn, DFd)	F (4, 15) = 38.89			
	**Bonferroni's multiple comparisons test**	**Mean Diff.**	**95.00% CI of diff.**	**Significant?**	**Summary**	**Adjusted P Value**
	TG (100 nM) vs. Control	-10.33	-12.85 to -7.802	Yes	****	<0.0001
	THA (100 µM) vs. Control	-4.042	-6.566 to -1.518	Yes	**	0.0016
	THA (50 µM) vs. Control	-3.002	-5.526 to -0.4774	Yes	*	0.0167
	THA (25 µM) vs. Control	-1.836	-4.360 to 0.6881	No	ns	0.2274

Fig. 1D	ANOVA table	F (DFn, DFd)	F (5, 18) = 22.16			
	**Bonferroni's multiple comparisons test**	**Mean Diff.**	**95.00% CI of diff.**	**Significant?**	**Summary**	**Adjusted P Value**
	TG (100 nM) vs. Control	-8.824	-12.09 to -5.554	Yes	****	<0.0001
	B3C (20 µM) vs. Control	-3.695	-6.965 to -0.4242	Yes	*	0.0221
	LBT (10 µM) vs. Control	-10.59	-13.86 to -7.317	Yes	****	<0.0001
	DAS (20 µM) vs. Control	-5.217	-8.487 to -1.946	Yes	**	0.0011
	DAC(20 µM)vs. Control	-4.49	-7.761 to -1.220	Yes	**	0.0047

Fig. 1E	ANOVA table	F (DFn, DFd)	F (4, 15) = 11.03			
	**Bonferroni's multiple comparisons test**	**Mean Diff.**	**95.00% CI of diff.**	**Significant?**	**Summary**	**Adjusted P Value**
	TG (100 nM) vs. Control	-1.016	-1.545 to -0.4868	Yes	***	0.0003
	THA (100 µM) vs. Control	-0.77	-1.299 to -0.2410	Yes	**	0.0036
	THA (50 µM) vs. Control	-0.5387	-1.068 to -0.009690	Yes	*	0.045
	THA (25 µM) vs. Control	-0.07772	-0.6067 to 0.4512	No	ns	>0.9999

Fig. 2A	ANOVA table	F (DFn, DFd)	F (12, 39) = 25.90			
	**Bonferroni's multiple comparisons test**	**Mean Diff.**	**95.00% CI of diff.**	**Significant?**	**Summary**	**Adjusted P Value**
	TG (100 nM) with TRE vs. without TRE	4.229	0.6941 to 7.764	Yes	**	0.0094
	THA (100 µM) with TRE vs. without TRE	4.933	1.398 to 8.468	Yes	**	0.0016
	B3C (20 µM) with TRE vs. without TRE	4.333	0.7977 to 7.868	Yes	**	0.0073
	LBT (10 µM)with TRE vs. without TRE	3.809	0.2744 to 7.345	Yes	*	0.0263
	DAS (20 µM) with TRE vs. without TRE	4.094	0.5585 to 7.629	Yes	*	0.0132
	DAC(20 µM) with TRE vs. without TRE	3.736	0.2010 to 7.271	Yes	*	0.0313
	TG (100 nM) vs. Control	-13.25	-16.79 to -9.718	Yes	****	<0.0001
	THA (100 µM) vs. Control	-5.655	-9.190 to -2.120	Yes	***	0.0002
	B3C (20 µM) vs. Control	-4.72	-8.255 to -1.185	Yes	**	0.0027
	LBT (10 µM) vs. Control	-9.223	-12.76 to -5.688	Yes	****	<0.0001
	DAS (20 µM) vs. Control	-4.335	-7.870 to -0.8001	Yes	**	0.0072
	DAC(20 µM)vs. Control	-3.969	-7.504 to -0.4338	Yes	*	0.0179

Fig. 2B	ANOVA table	F (DFn, DFd)	F (4, 15) = 21.62			
	**Bonferroni's multiple comparisons test**	**Mean Diff.**	**95.00% CI of diff.**	**Significant?**	**Summary**	**Adjusted P Value**
	TG (100 nM) vs. Control	-1.243	-1.938 to -0.5481	Yes	***	0.0005
	THA (100 µM) vs. Control	-2.123	-2.818 to -1.428	Yes	****	<0.0001
	TG (100 nM) with TRE vs. without TRE	0.7291	0.03434 to 1.424	Yes	*	0.0376
	THA (100 µM) with TRE vs. without TRE	1.377	0.6828 to 2.072	Yes	***	0.0002

Fig. 2C	ANOVA table	F (DFn, DFd)	F (5, 36) = 60.90			
	**Bonferroni's multiple comparisons test**	**Mean Diff.**	**95.00% CI of diff.**	**Significant?**	**Summary**	**Adjusted P Value**
	Bt_2-cAMP (1mM) vs. Control	-122.2	-147.3 to -97.10	Yes	****	<0.0001
	TG (100 nM) vs. Control	-39.24	-64.34 to -14.14	Yes	***	0.0007
	THA (100 µM) vs. Control	-32.8	-57.90 D36 to -7.703	Yes	**	0.0054
	TG (100 nM) with TRE vs. without TRE	46.85	21.75 to 71.95	Yes	****	<0.0001
	THA (100 µM) with TRE vs. without TRE	48.36	23.26 to 73.46	Yes	****	<0.0001

Fig. 2D	ANOVA table	F (DFn, DFd)	F (4, 20) = 54.59			
	**Bonferroni's multiple comparisons test**	**Mean Diff.**	**95.00% CI of diff.**	**Significant?**	**Summary**	**Adjusted P Value**
	Group -Triton TG (100 nM) vs. Control	0.6888	0.5336 to 0.8440	Yes	****	<0.0001
	Group -Triton THA (100 µM) vs. Control	0.7166	0.5614 to 0.8718	Yes	****	<0.0001
	Group -Triton TG (100 nM) with TRE vs.	-0.3806	-0.5358 to -0.2254	Yes	****	<0.0001
	Group -Triton THA (100 µM) with TRE vs. without TRE	-0.267	-0.4222 to -0.1118	Yes	***	0.0005

Fig. 4C	ANOVA table	F (DFn, DFd)	F (4, 15) = 28.29			
	**Bonferroni's multiple comparisons test**	**Mean Diff.**	**95.00% CI of diff.**	**Significant?**	**Summary**	**Adjusted P Value**
	TG (100 nM) vs. Control	-13.85	‘-17.94 to -9.748	Yes	****	<0.0001
	THA (100 µM) vs. Control	-6.936	-11.03 to -2.838	Yes	***	0.0009
	THA (50 µM) vs. Control	-4.768	-8.866 to -0.6697	Yes	*	0.0194
	THA (25 µM) vs. Control	-1.55	-5.648 to 2.549	No	ns	>0.9999

Fig. 4D	ANOVA table	F (DFn, DFd)	F (4, 15) = 51.47			
	**Bonferroni's multiple comparisons test**	**Mean Diff.**	**95.00% CI of diff.**	**Significant?**	**Summary**	**Adjusted P Value**

	TG (100 nM) vs. Control	-17.89	-21.74 to -14.03	Yes	****	<0.0001
	THA (100 µM) vs. Control	-6.814	-10.67 to -2.960	Yes	***	0.0006
	TG (100 nM) with TRE vs. without TRE	12.54	8.690 to 16.40	Yes	****	<0.0001
	THA (100 µM) with TRE vs. without TRE	4.39	0.5367 to 8.244	Yes	*	0.0224

**Fig. 4E**	**ANOVA table**	F (DFn, DFd)	F (4, 15) = 17.69			
	**Bonferroni's multiple comparisons test**	**Mean Diff.**	**95.00% CI of diff.**	**Significant?**	**Summary**	**Adjusted P Value**
	TG (100 nM) vs. Control	-1.527	-2.384 to -0.6707	Yes	***	0.0006
	THA (100 µM) vs. Control	-2.208	-3.064 to -1.351	Yes	****	<0.0001
	THA (50 µM) vs. Control	-0.6807	-1.537 to 0.1758	No	ns	0.1582
	THA (25 µM) vs. Control	-0.3716	-1.228 to 0.4849	No	ns	0.9494

**Fig. 4F**	**ANOVA table**	F (DFn, DFd)	F (4, 15) = 17.05			
	**Bonferroni's multiple comparisons test**	**Mean Diff.**	**95.00% CI of diff.**	**Significant?**	**Summary**	**Adjusted P Value**
	TG (100 nM) vs. Control	-1.573	-2.252 to -0.8947	Yes	****	<0.0001
	THA (100 µM) vs. Control	-1.746	-2.425 to -1.068	Yes	****	<0.0001
	TG (100 nM) with TRE vs. without TRE	0.8269	0.1485 to 1.505	Yes	*	0.0141
	THA (100 µM) with TRE vs. without TRE	0.6955	0.01707 to 1.374	Yes	*	0.0433

**Fig. 5C**	**ANOVA table**	F (DFn, DFd)	F (4, 15) = 15.65			
	**Bonferroni's multiple comparisons test**	**Mean Diff.**	**95.00% CI of diff.**	**Significant?**	**Summary**	**Adjusted P Value**
	TG (100 nM) vs. Control	-1.707	-2.430 to -0.9841	Yes	****	<0.0001
	THA (100 µM) vs. Control	-1.181	-1.904 to -0.4582	Yes	**	0.0013
	TG (100 nM) with TRE vs. without TRE	1.236	0.5130 to 1.959	Yes	***	0.0008
	THA (100 µM) with TRE vs. without TRE	0.9772	0.2543 to 1.700	Yes	**	0.0065

**Fig. 6C**	**ANOVA table**	F (DFn, DFd)	F (5, 18) = 26.12			
	**Bonferroni's multiple comparisons test**	**Mean Diff.**	**95.00% CI of diff.**	**Significant?**	**Summary**	**Adjusted P Value**
	TG (100 nM) vs. Control	-2.291	-2.910 to -1.671	Yes	****	<0.0001
	GAL (10 µM) vs. Control	-0.4519	-1.071 to 0.1673	No	ns	0.25
	THA (100 µM) vs. Control	-0.9208	-1.540 to -0.3016	Yes	**	0.0023
	THA (50 µM) vs. Control	-0.7265	-1.346 to -0.1073	Yes	*	0.0168
	THA (25 µM) vs. Control	-0.6354	-1.255 to -0.01620	Yes	*	0.0425

**Fig. 6D**	**ANOVA table**	F (DFn, DFd)	F (4, 15) = 33.73			
	**Bonferroni's multiple comparisons test**	**Mean Diff.**	**95.00% CI of diff.**	**Significant?**	**Summary**	**Adjusted P Value**
	TG (100 nM) vs. Control	-4.23	-5.438 to -3.023	Yes	****	<0.0001
	THA (100 µM) vs. Control	-2.129	-3.337 to -0.9212	Yes	***	0.0006
	TG (100 nM) with TRE vs. without TRE	2.919	1.711 to 4.126	Yes	****	<0.0001
	THA (100 µM) with TRE vs. without TRE	2.079	0.8716 to 3.287	Yes	***	0.0008

**Fig. 6E**	**ANOVA table**	F (DFn, DFd)	F (4, 15) = 25.88			
	**Bonferroni's multiple comparisons test**	**Mean Diff.**	**95.00% CI of diff.**	**Significant?**	**Summary**	**Adjusted P Value**
	TG (100 nM) vs. Control	-1.271	-1.723 to -0.8190	Yes	****	<0.0001
	THA (100 µM) vs. Control	-0.5116	-0.9638 to -0.05940	Yes	*	0.0234
	THA (50 µM) vs. Control	-0.1533	-0.6055 to 0.2989	No	ns	>0.9999
	THA (25 µM) vs. Control	0.1787	-0.2736 to 0.6309	No	ns	>0.9999

**Fig. 6F**	**ANOVA table**	F (DFn, DFd)	F (4, 15) = 12.19			
	**Bonferroni's multiple comparisons test**	**Mean Diff.**	**95.00% CI of diff.**	**Significant?**	**Summary**	**Adjusted P Value**
	TG (100 nM) vs. Control	-2.17	-3.157 to -1.184	Yes	****	<0.0001
	THA (100 µM) vs. Control	-1.256	-2.242 to -0.2694	Yes	*	0.0103
	TG (100 nM) with TRE vs. without TRE	1.211	0.2245 to 2.197	Yes	*	0.0134
	THA (100 µM) with TRE vs. without TRE	0.9876	0.001120 to 1.974	Yes	*	0.0497

**Fig. 6H**	**ANOVA table**	F (DFn, DFd)	F (4, 15) = 18.63			
	**Bonferroni's multiple comparisons test**	**Mean Diff.**	**95.00% CI of diff.**	**Significant?**	**Summary**	**Adjusted P Value**
	TG (100 nM) vs. Control	0.7232	0.4600 to 0.9863	Yes	****	<0.0001
	THA (100 µM) vs. Control	0.6174	0.3543 to 0.8806	Yes	****	<0.0001
	TG (100 nM) with TRE vs. without TRE	-0.3974	-0.6606 to -0.1343	Yes	**	0.0027
	THA (100 µM) with TRE vs. without TRE	-0.2922	-0.5554 to -0.02909	Yes	*	0.0267

**Fig. 7C**	**ANOVA table**	F (DFn, DFd)	F (4, 15) = 124.9			
	**Bonferroni's multiple comparisons test**	**Mean Diff.**	**95.00% CI of diff.**	**Significant?**	**Summary**	**Adjusted P Value**
	TG (100 nM) vs. Control	-14.98	-17.28 to -12.69	Yes	****	<0.0001
	THA (100 µM) vs. Control	-2.443	-4.737 to -0.1499	Yes	*	0.0343
	THA (50 µM) vs. Control	-0.9414	-3.235 to 1.352	No	ns	>0.9999
	THA (25 µM) vs. Control	-0.07203	-2.366 to 2.221	No	ns	>0.9999

**Fig. 7D**	**ANOVA table**	F (DFn, DFd)	F (4, 15) = 117.1			
	**Bonferroni's multiple comparisons test**	**Mean Diff.**	**95.00% CI of diff.**	**Significant?**	**Summary**	**Adjusted P Value**
	TG (100 nM) vs. Control	-15.47	-17.86 to -13.09	Yes	****	<0.0001

	THA (100 μM) vs. Control	-2.815	-5.199 to -0.4323	Yes	*	0.0175
	TG (100 nM) with TRE vs. without TRE	10.97	8.584 to 13.35	Yes	****	<0.0001
	THA (100 μM) with TRE vs. without TRE	2.989	0.6056 to 5.372	Yes	*	0.0115

**Fig. 7F**	**ANOVA table**	F (DFn, DFd)	F (4, 15) = 11.40			
	**Bonferroni's multiple comparisons test**	**Mean Diff.**	**95.00% CI of diff.**	**Significant?**	**Summary**	**Adjusted P Value**
	Group -Triton TG (100 nM) vs. Control	0.7163	0.3252 to 1.107	Yes	***	0.0004
	Group -Triton THA (100 μM) vs. Control	0.4594	0.06838 to 0.8505	Yes	*	0.0182
	Group -Triton TG (100 nM) with TRE vs.	-0.5064	-0.8975 to -0.1154	Yes	**	0.009
	Group -Triton THA (100 μM) with TRE vs. without TRE	-0.539	-0.9300 to -0.1479	Yes	**	0.0056

**Fig. 8**	**Welch's *t* -test**	
	**P Value**	0.0227
	**Summary**	*
	**Are means signif. different? (P < 0.05)**	Yes
	**One- or two-tailed P value?**	Two-tailed
	**Welch correction，t，df**	t=6.526, df=2.000

Supplementary_Material_1 Western blotting

Fig. 1A

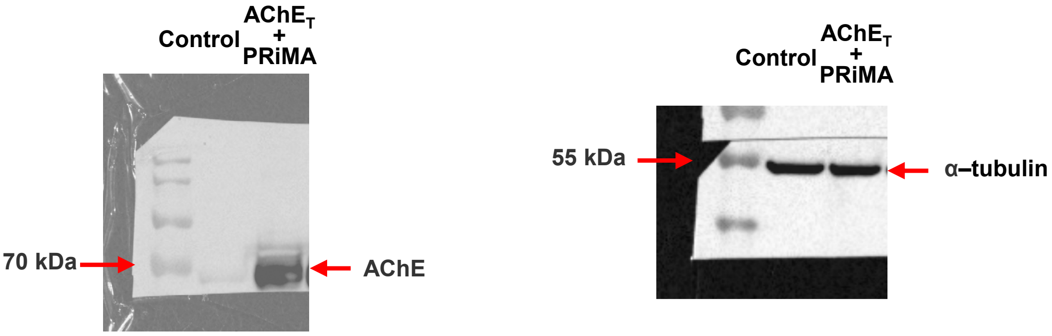

Fig. 1C

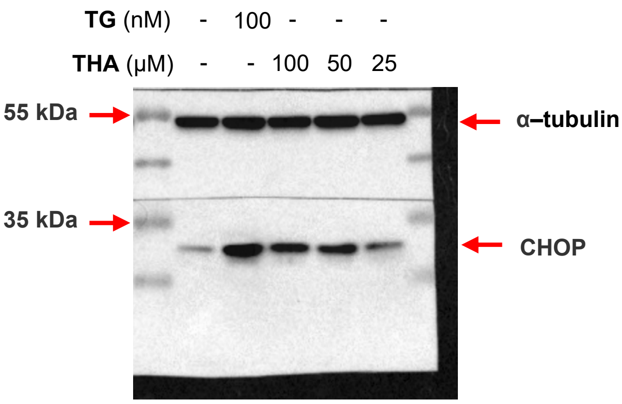

Fig. 1D

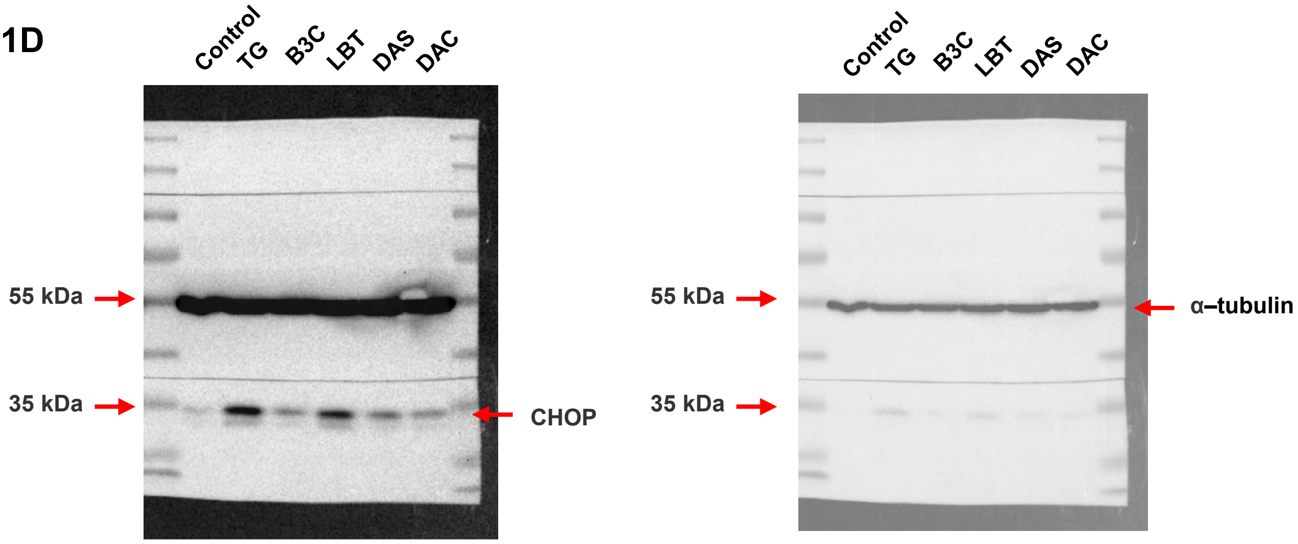

Fig. 1E

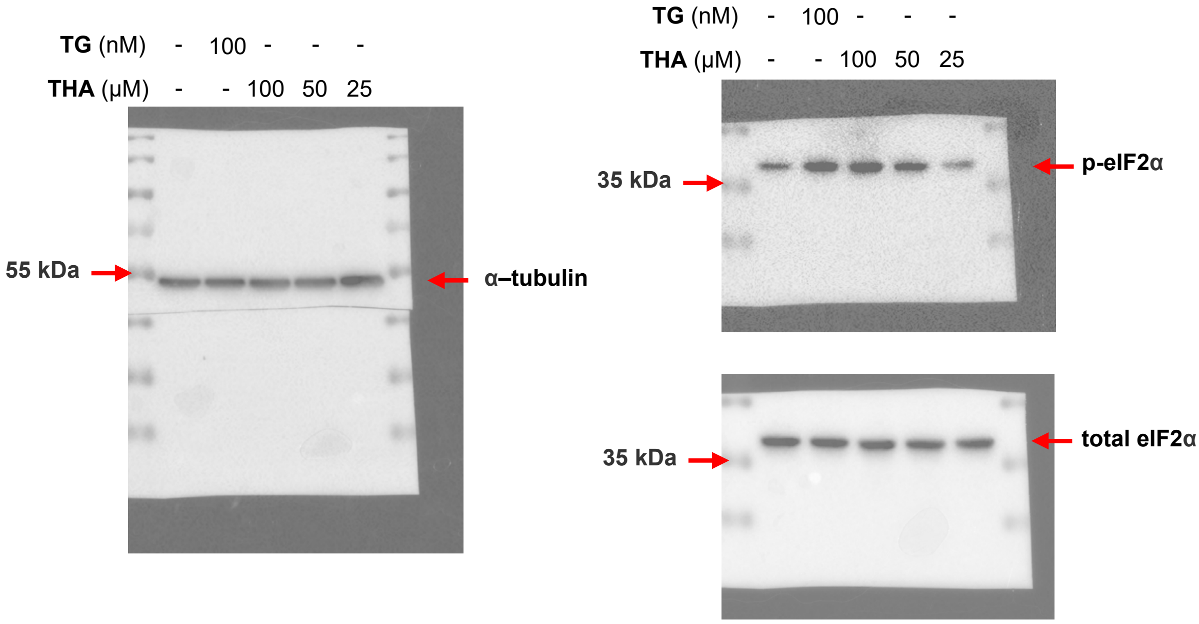

Fig. 2A

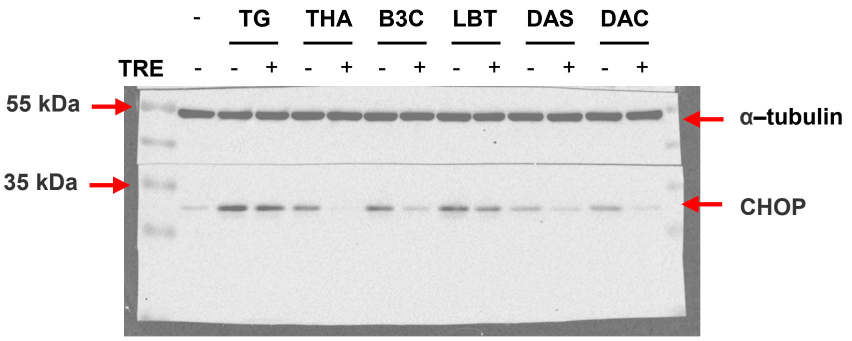
- TG THA B3C LBT DAS DAC
TRE - - + - + - + - + - + - +
55 kDa
35 kDa
α–tubulin
CHOP

Fig. 2B

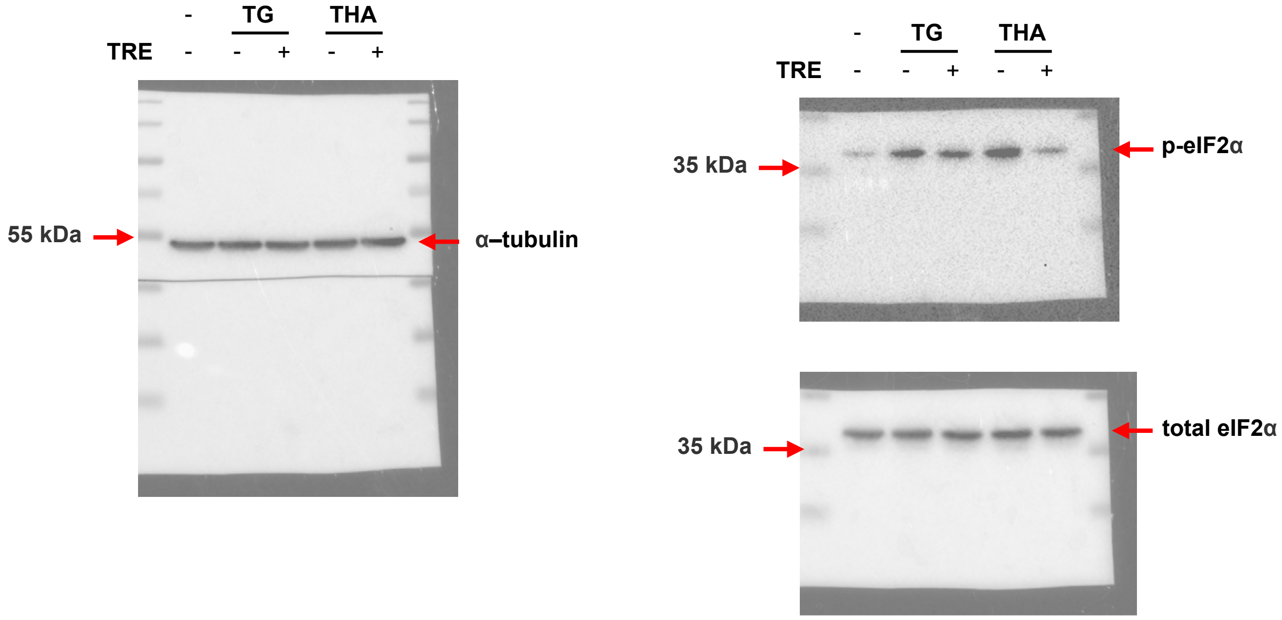
- TG THA
TRE - - + - +
55 kDa
α–tubulin
- TG THA
TRE - - + - +
35 kDa
p-eIF2α
35 kDa
total eIF2α

Fig. 3

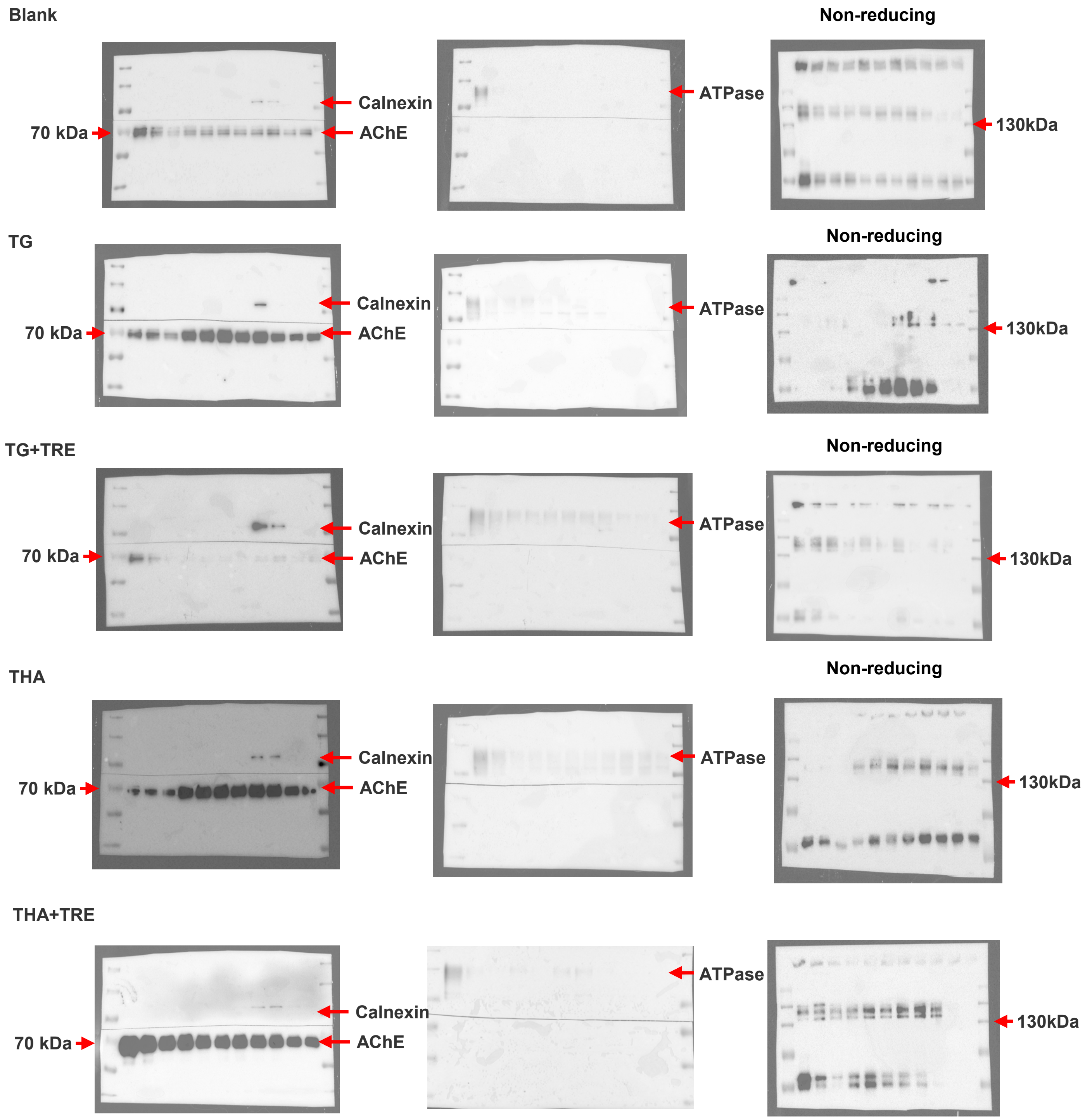
Blank
Non-reducing
Calnexin
70 kDa
AChE
ATPase
130kDa
TG
Non-reducing
Calnexin
70 kDa
AChE
ATPase
130kDa
TG+TRE
Non-reducing
Calnexin
70 kDa
AChE
ATPase
130kDa
THA
Non-reducing
Calnexin
70 kDa
AChE
ATPase
130kDa
THA+TRE
Calnexin
70 kDa
AChE
ATPase
130kDa

Fig. 4A

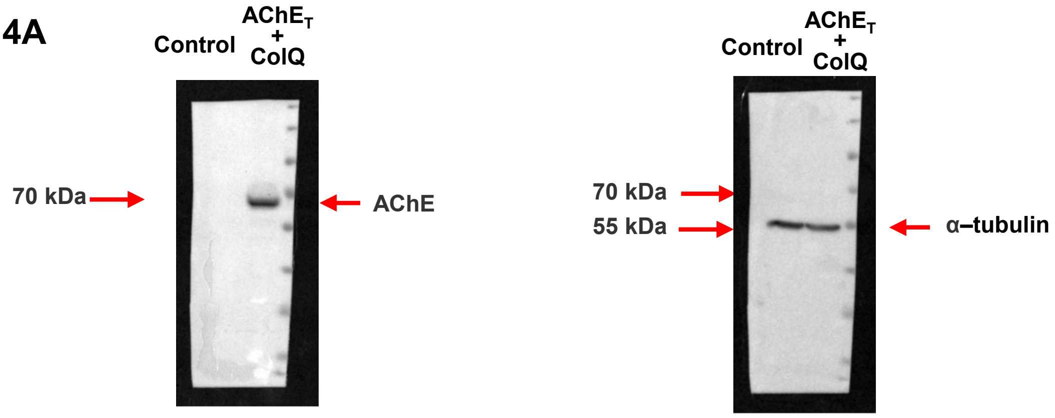

Fig. 4C

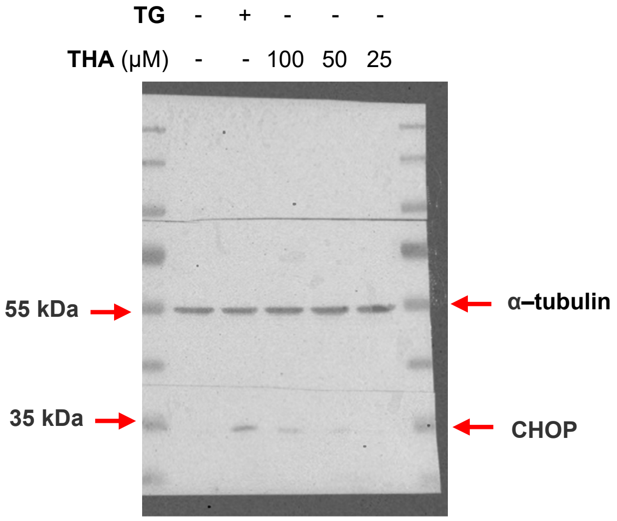

Fig. 4D

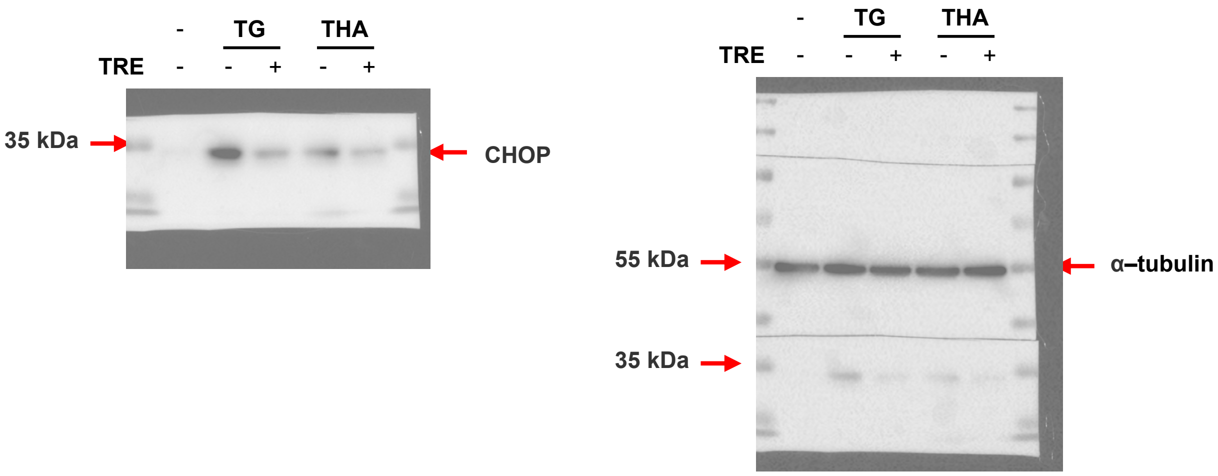

Fig. 4E

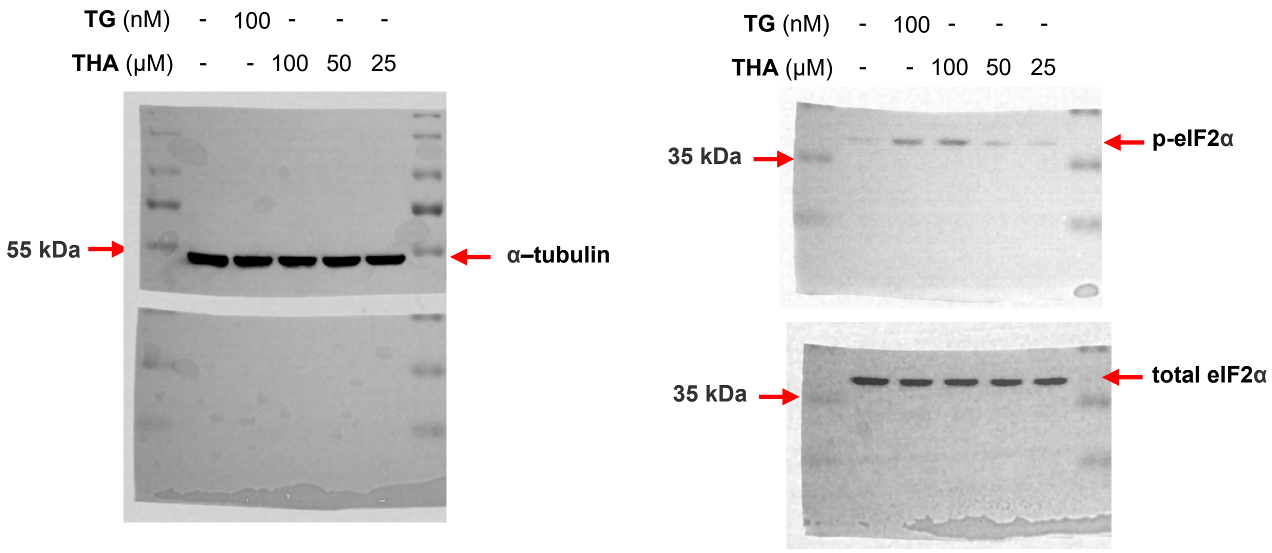

TG (nM) - 100 - - -
THA (μM) - - 100 50 25
55 kDa
α–tubulin
TG (nM) - 100 - - -
THA (μM) - - 100 50 25
35 kDa
p-eIF2α
35 kDa
total eIF2α

Fig. 4F

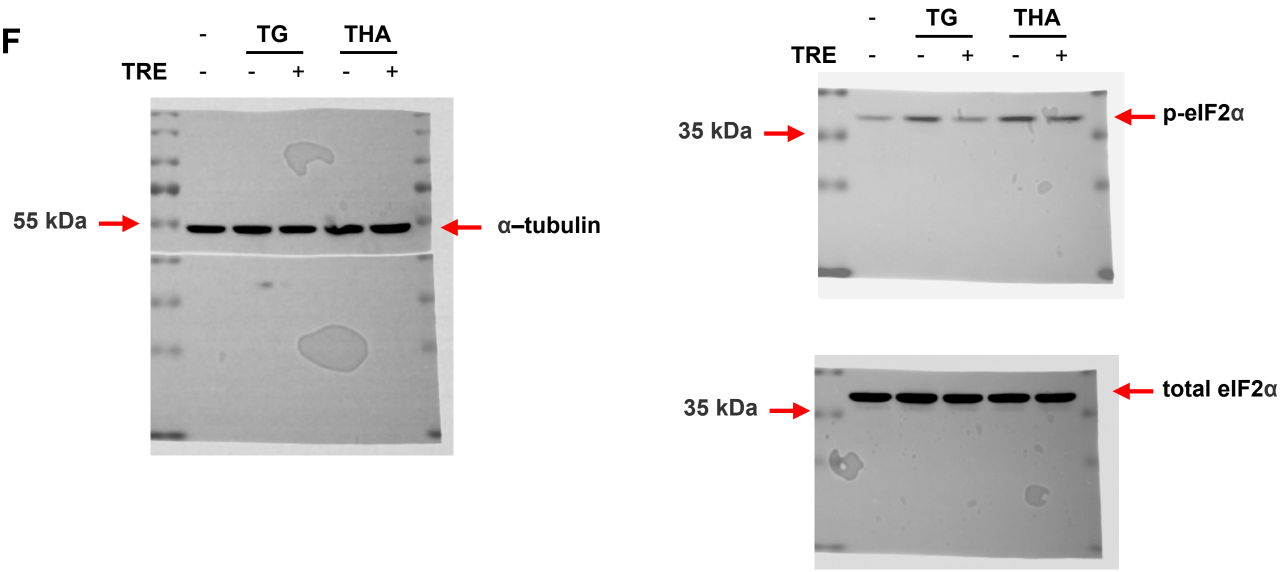

- TG THA
TRE - - + - +
55 kDa
α–tubulin
- TG THA
TRE - - + - +
35 kDa
p-eIF2α
35 kDa
total eIF2α

Fig. 5A

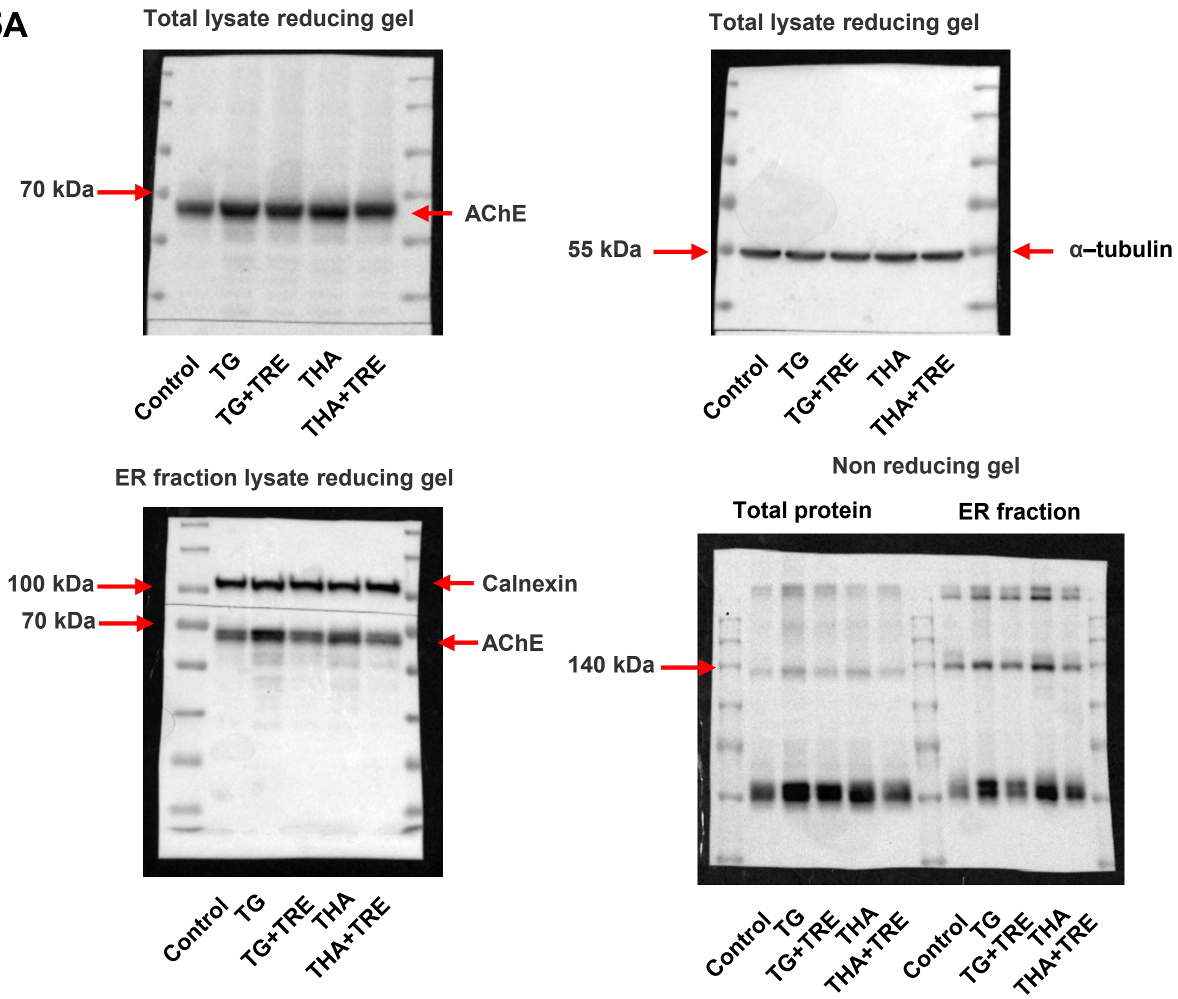
Total lysate reducing gel
70 kDa
AChE
Control
TG
TG+TRE
THA
THA+TRE
Total lysate reducing gel
55 kDa
α–tubulin
Control
TG
TG+TRE
THA
THA+TRE
ER fraction lysate reducing gel
100 kDa
70 kDa
Calnexin
AChE
Control
TG
TG+TRE
THA
THA+TRE
Non reducing gel
Total protein
ER fraction
140 kDa
Control
TG
TG+TRE
THA
THA+TRE
Control
TG
TG+TRE
THA
THA+TRE

Fig. 6A

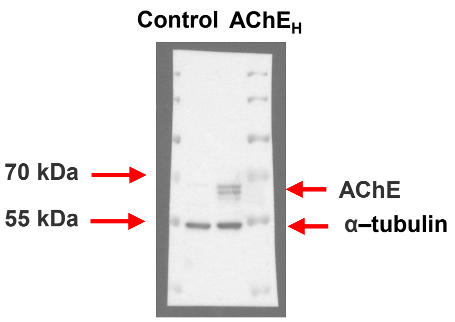

Fig. 6C

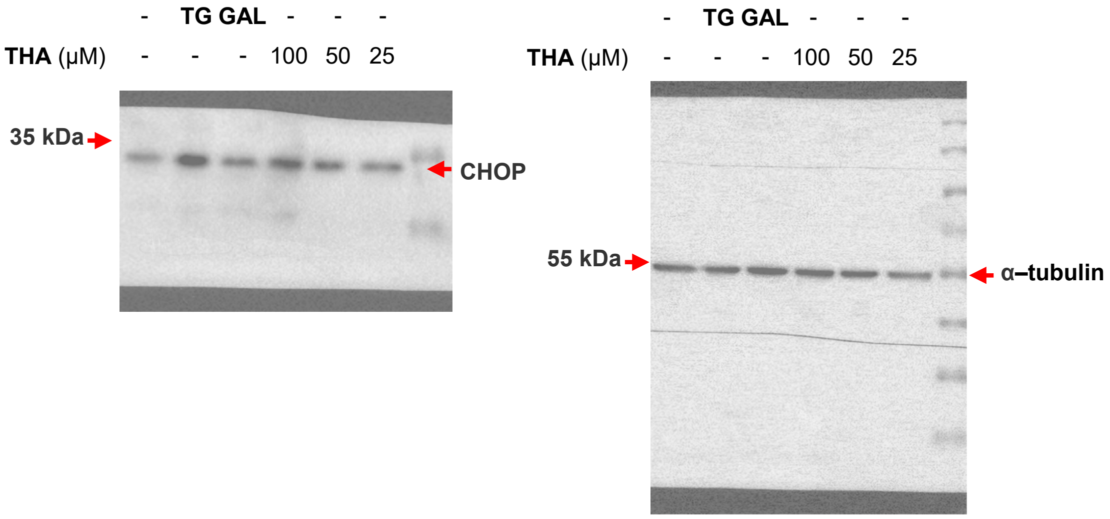

Fig. 6D

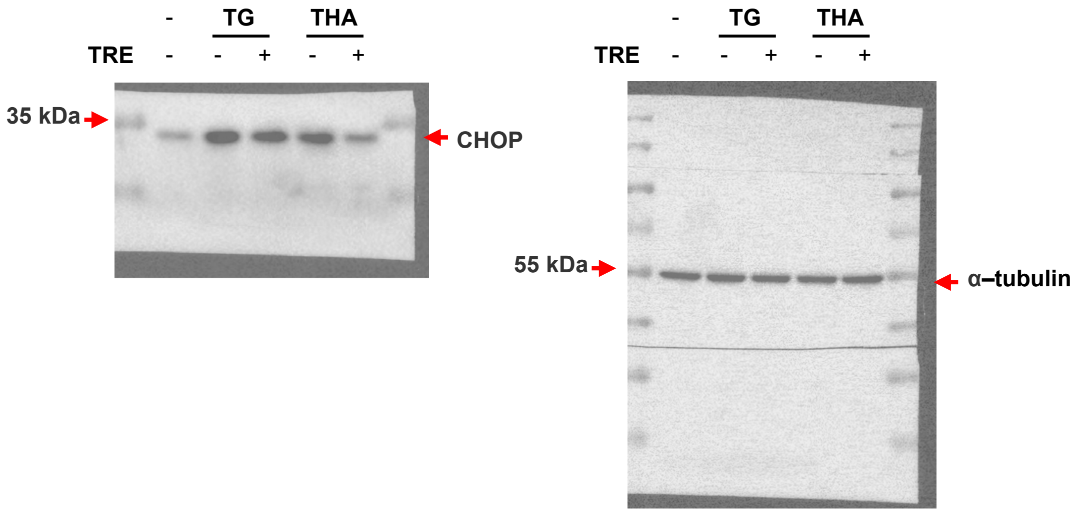

Fig. 6E

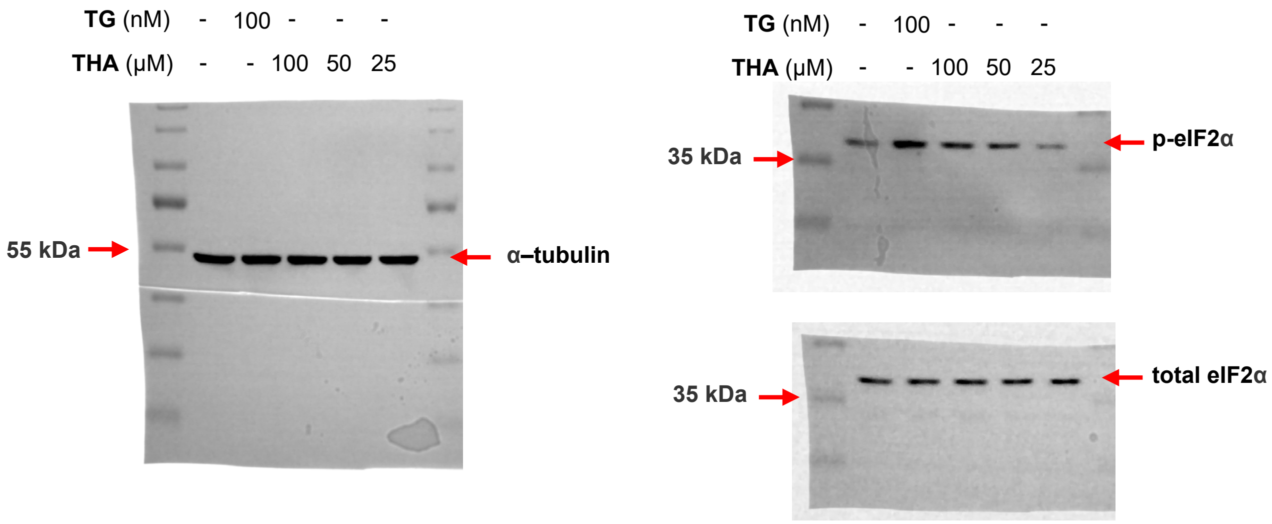

TG (nM) - 100 - - -
THA (μM) - - 100 50 25
55 kDa
α–tubulin
TG (nM) - 100 - - -
THA (μM) - - 100 50 25
35 kDa
p-eIF2α
35 kDa
total eIF2α

Fig. 6F

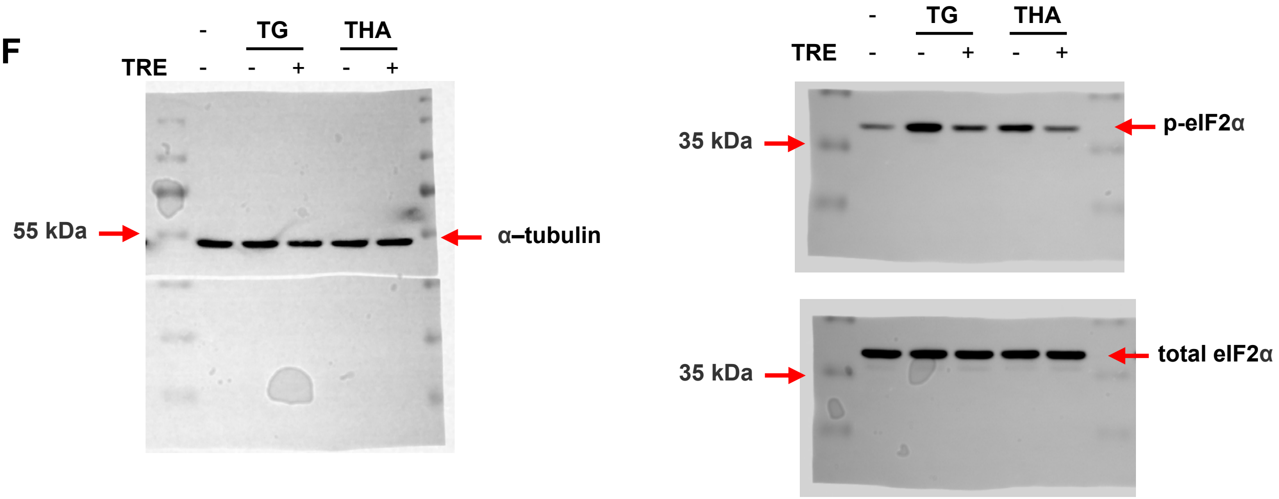

- TG THA
TRE - - + - +
55 kDa
α–tubulin
- TG THA
TRE - - + - +
35 kDa
p-eIF2α
35 kDa
total eIF2α

Fig. 7A

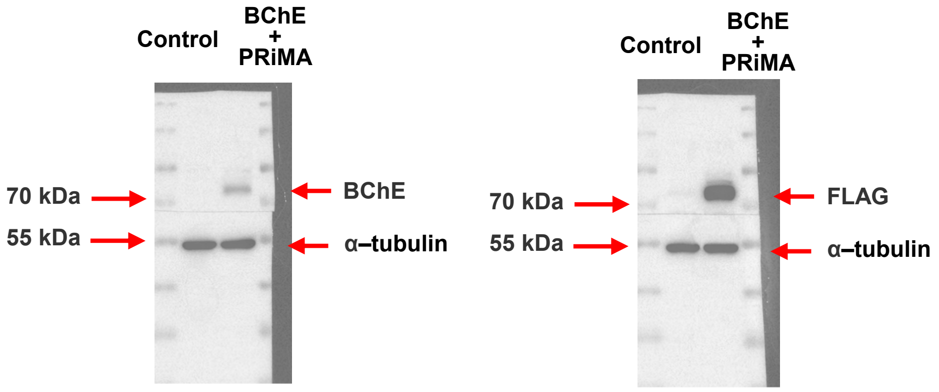

Fig. 7C

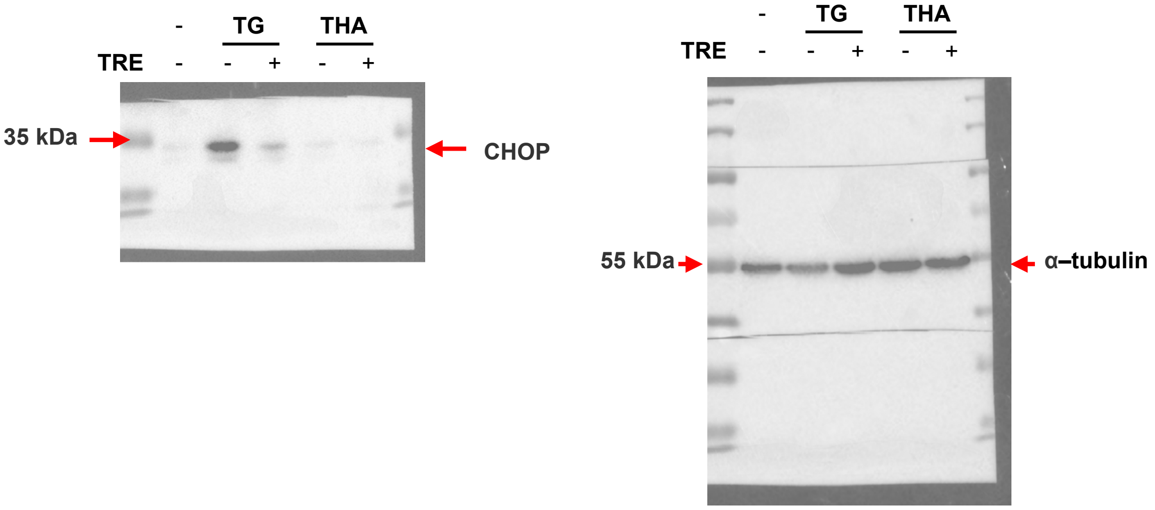

Fig. 7D

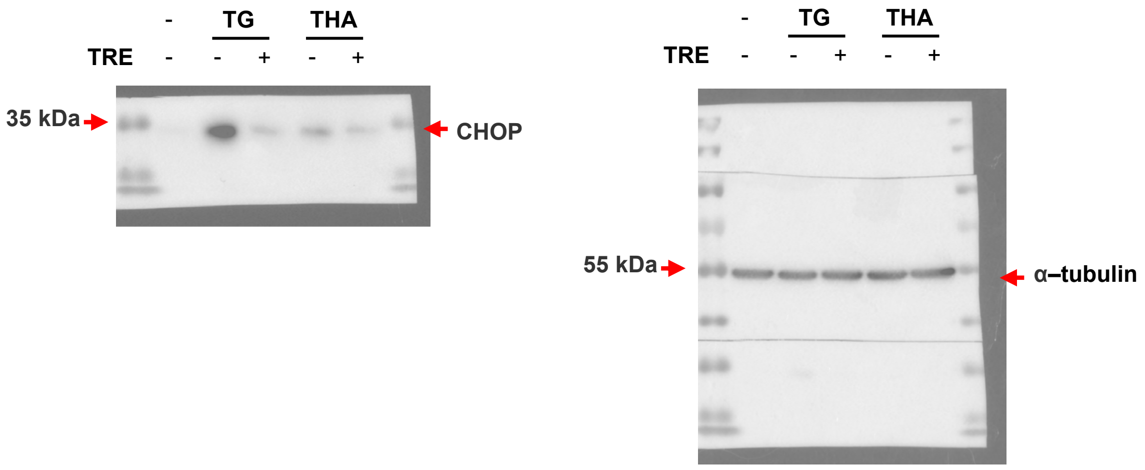

Fig. S1

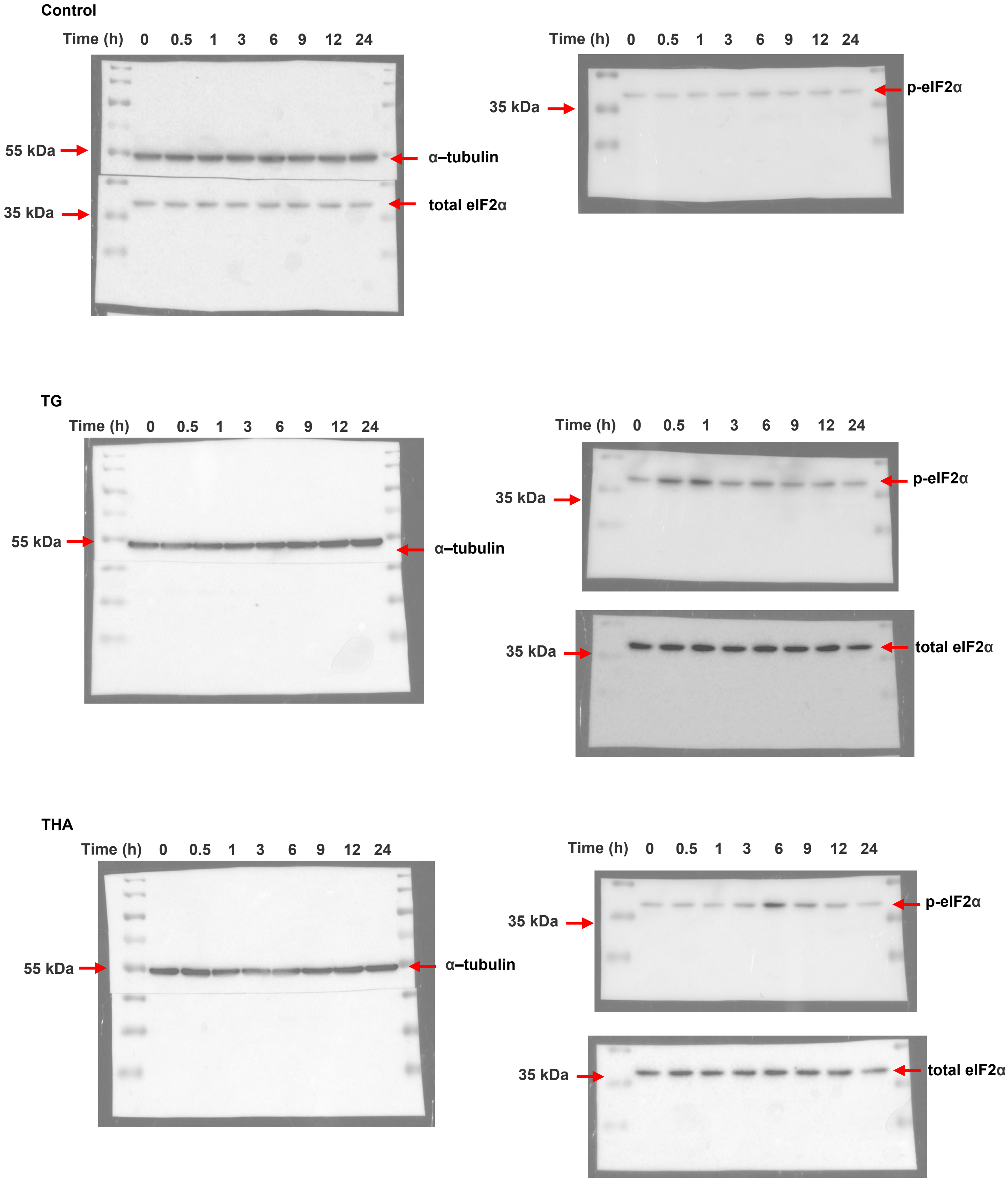
Control
Time (h) 0 0.5 1 3 6 9 12 24
55 kDa
35 kDa
α–tubulin
total eIF2α
Time (h) 0 0.5 1 3 6 9 12 24
35 kDa
p-eIF2α
TG
Time (h) 0 0.5 1 3 6 9 12 24
55 kDa
α–tubulin
Time (h) 0 0.5 1 3 6 9 12 24
35 kDa
p-eIF2α
35 kDa
total eIF2α
THA
Time (h) 0 0.5 1 3 6 9 12 24
55 kDa
α–tubulin
Time (h) 0 0.5 1 3 6 9 12 24
35 kDa
p-eIF2α
35 kDa
total eIF2α

Fig. S2

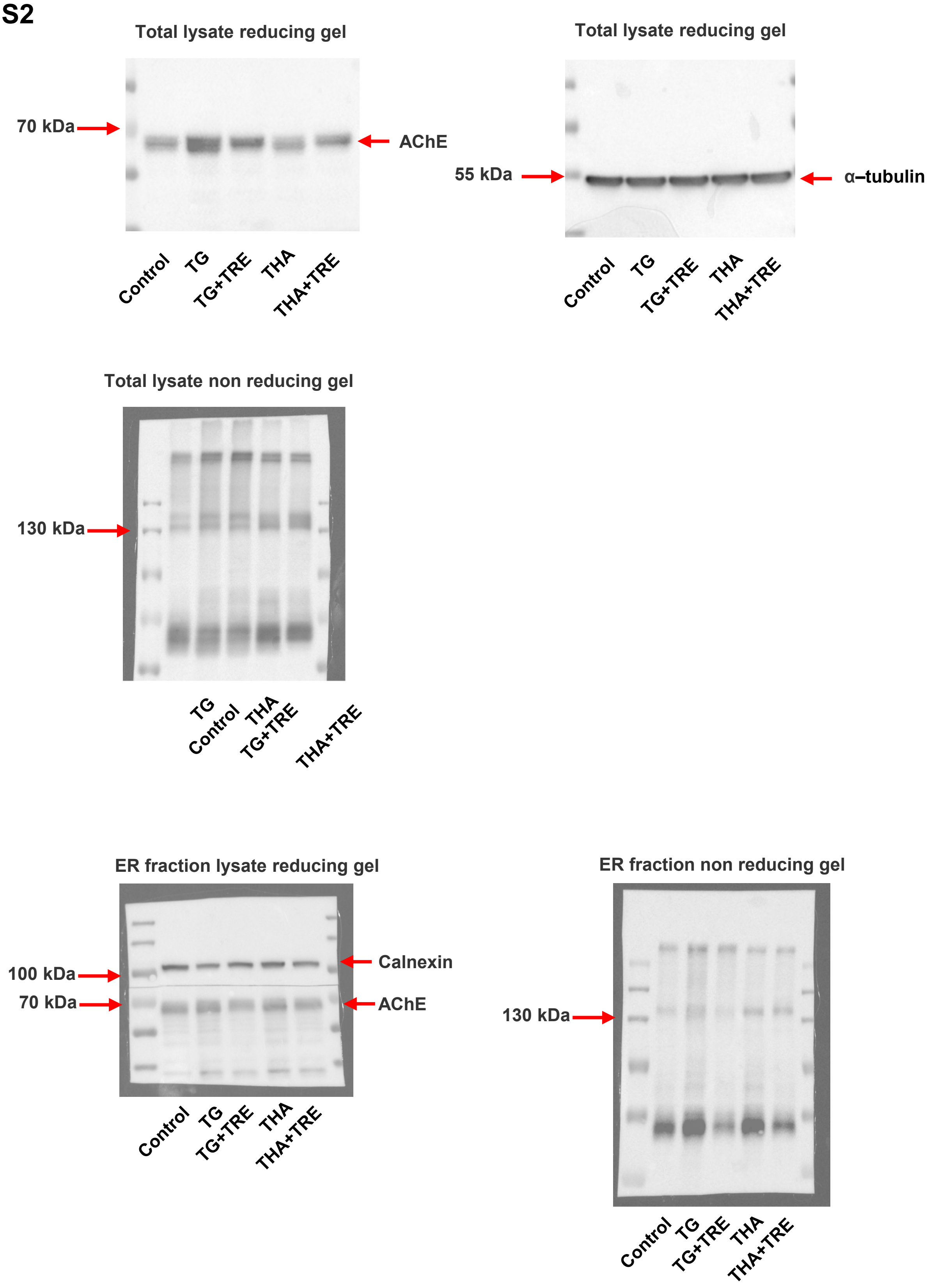
Total lysate reducing gel
70 kDa
AChE
Control
TG
TG+TRE
THA
THA+TRE
Total lysate reducing gel
55 kDa
α–tubulin
Control
TG
TG+TRE
THA
THA+TRE
Total lysate non reducing gel
130 kDa
TG
Control
THA
TG+TRE
THA+TRE
ER fraction lysate reducing gel
100 kDa
70 kDa
Calnexin
AChE
Control
TG
TG+TRE
THA
THA+TRE
ER fraction non reducing gel
130 kDa
Control
TG
TG+TRE
THA
THA+TRE
